# Catestatin restores cardiac metabolic flexibility and enhances mitochondrial function

**DOI:** 10.1038/s42003-026-10310-z

**Published:** 2026-05-23

**Authors:** Satadeepa Kal, Venkat R. Chirasani, Nilima Biswas, Sumana Mahata, Suborno Jati, Kechun Tang, Teresa Pasqua, Ennio Avolio, Ramamurthy Chitteti, Shrabastee Chakraborty, Gautam Bandyopadhyay, Brian P. Head, Geert van den Bogaart, Hemal H. Patel, Debashis Sahoo, Sanjib Senapati, Sushil K. Mahata

**Affiliations:** 1 Veterans Medical Research Foundation, San Diego, CA USA; 2 University of California San Diego, La Jolla, CA USA; 3 Department of Biotechnology, Indian Institute of Technology Madras, Chennai, India; 4 Riverside University Health System, Riverside, CA USA; 5 University Magna Graecia of Catanzaro, Catanzaro, Italy; 6 University of Calabria, Cosenza, Italy; 7 VA San Diego Healthcare System, San Diego, CA USA; 8 University of Michigan, Ann Arbor, MI USA; 9 University of Groningen, Groningen, The Netherlands; 10 Present Address: Department of Biochemistry and Biophysics, R. L. Juliano Structural Bioinformatics Core, University of North Carolina at Chapel Hill, Chapel Hill, NC USA

**Keywords:** Metabolic syndrome, Experimental models of disease, Diabetes

## Abstract

Hypertension is a major risk factor for heart failure, characterized by impaired energy metabolism and mitochondrial dysfunction. The endogenous peptide catestatin (CST) has known cardiovascular protective effects, but its role in cardiac metabolism remains unclear. Here, we show that CST regulates cardiac metabolic pathways through integrated transcriptomic and network analyses, identifying cell-type-specific gene programs that are disrupted in its absence and restored with supplementation. Comparative analysis with human heart failure datasets reveals conserved alterations in glucose and fatty acid metabolism and mitochondrial function. Functional studies demonstrate that CST restores metabolic flexibility by shifting substrate utilization toward glucose oxidation. Mechanistically, CST enhances mitochondrial ATP production by interacting with ATP synthase and improving membrane potential and enzyme activity. These findings establish CST as a key regulator of cardiac energy metabolism and reveal an endocrine–mitochondrial signaling axis with therapeutic potential for hypertension-associated heart failure.

**Figure:**
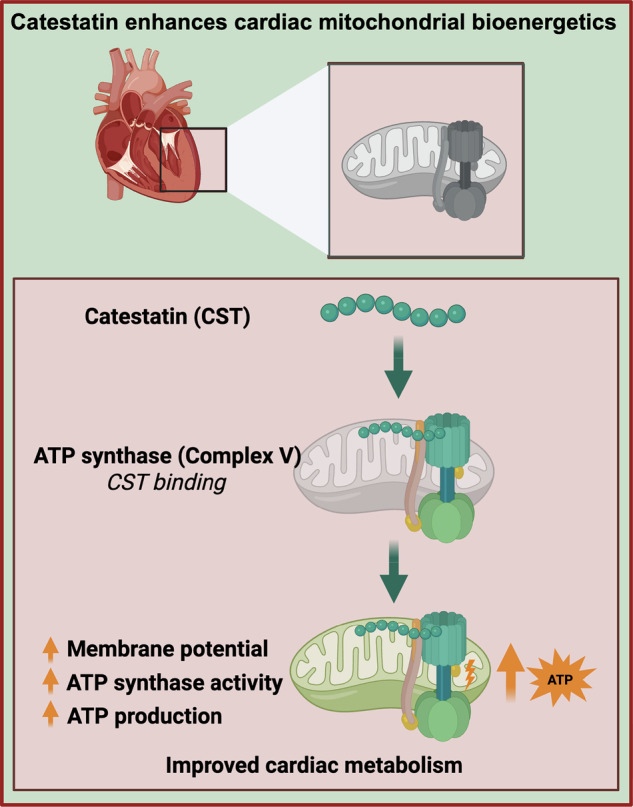

## Abstract (web summary)

Hypertension drives heart failure through metabolic and mitochondrial dysfunction. Catestatin restores metabolic flexibility in CSTKO hearts, shifts fuel use, and enhances ATP synthase activity, revealing a therapeutic endocrine–mitochondrial axis.

## Introduction

The heart has one of the highest energy demands of any organ, requiring continuous production of adenosine triphosphate (ATP) to sustain contractile and ionic function^1–5^. In the adult myocardium, more than 95% of ATP is generated through mitochondrial oxidative phosphorylation (OXPHOS)^6,7^. Because intracellular ATP stores are limited and turn over rapidly, uninterrupted energy production is essential for maintaining cardiac function and viability^4,8,9^. To meet this demand, the heart exhibits remarkable metabolic flexibility, oxidizing multiple substrates including fatty acids (FA)^6–8^, glucose^7,10,11^, lactate^7,11,12^ and, to a lesser extent, ketone bodies and amino acids^1,13–15^. This capacity to dynamically switch between substrates—termed metabolic flexibility^16,17^ - is a defining feature of healthy cardiac physiology. During acute stress or ischemia, substrate preference shifts from FA oxidation toward glucose oxidation to optimize oxygen efficiency. In conditions such as insulin resistance, however, metabolic flexibility is impaired, leading to excessive reliance on fatty acid oxidation, reduced energetic efficiency, and progressive cardiac dysfunction^16,18^.

Chromogranin A–derived catestatin (CST; hCgA_352–372_) is an endogenous peptide with established antihypertensive and cardioprotective properties^19–21^. CST improves insulin sensitivity in diet-induced obesity by reducing inflammation, alleviating endoplasmic reticulum stress, and suppressing hepatic gluconeogenesis^22,23^. Mice lacking CST develop insulin resistance even under normal dietary conditions and exhibit hypertension, cardiac hypertrophy, elevated catecholamines, and systemic inflammation, highlighting a critical role for CST in cardiometabolic homeostasis^24^. These observations suggest that CST may preserve cardiac function by maintaining metabolic flexibility within cardiomyocytes.

Insulin signaling plays a central role in regulating cardiac substrate utilization by promoting glucose uptake while restraining excessive fatty acid oxidation^15–17^. In insulin-resistant states, elevated circulating FA drives increased fatty acid uptake and oxidation while suppressing glucose utilization, resulting in a maladaptive metabolic shift that contributes to cardiac dysfunction^9,25^. Identifying mechanisms that restore balanced substrate utilization and improve mitochondrial efficiency is therefore essential for preventing cardiometabolic disease.

To define CST-dependent regulatory networks, we applied machine learning-based Boolean implication analysis^26,27^ to transcriptomic profiles from cardiomyocytes, fibroblasts, and macrophages—key cardiac cell types that coordinate metabolic and inflammatory responses. Cross-species integrative analysis of human, mouse, and rat datasets revealed that CST deficiency disrupts mitochondrial gene programs and pathways governing energy homeostasis. Complementary biochemical and imaging studies further demonstrate that CST localizes to mitochondria and interacts with F₁-ATP synthase, enhancing mitochondrial membrane potential, ATP synthase activity, and ATP production.

In this study, we identify CST as a regulator of cardiac metabolic homeostasis and mitochondrial function. Integrative transcriptomic and network analyses reveal CST-dependent gene programs that are disrupted in CST-deficient hearts and restored by CST supplementation, with parallel alterations observed in human heart failure (HF) datasets. Functional studies show that CST restores metabolic flexibility by rebalancing substrate utilization, reducing reliance on fatty acid oxidation, and enhancing glucose oxidation. Mechanistically, CST associates with mitochondrial ATP synthase, leading to increased membrane potential, enzyme activity, and ATP production. These findings establish a direct link between an endogenous peptide and mitochondrial energy metabolism in the heart and highlight a potential therapeutic axis for cardiometabolic dysfunction associated with hypertension.

## Results

### CST deficiency alters fibroblast and cardiomyocyte gene programs associated with HF

**Fig. 1:**
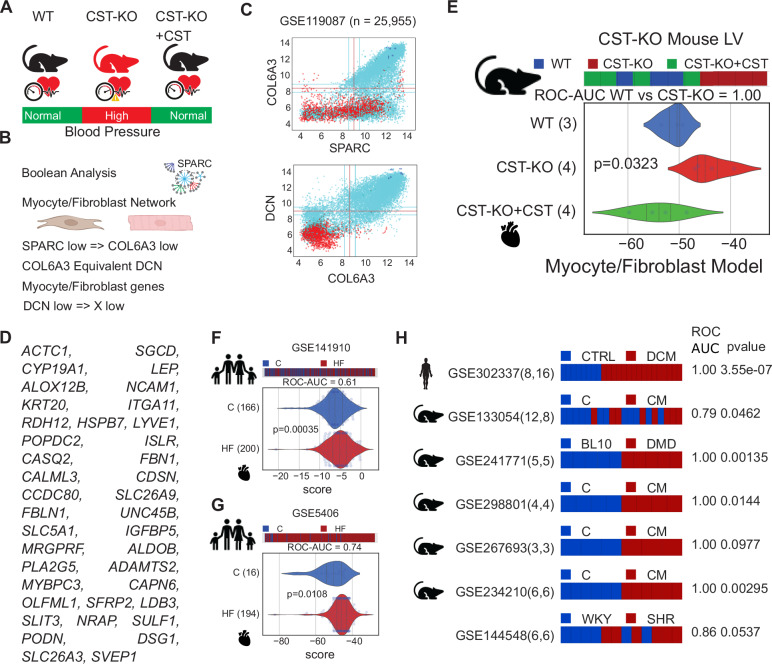
Boolean analysis of RNA-seq data identifies a CST-regulated MyoFibro gene signature. **A** Schematic illustrating the role of catestatin (CST) in the regulation of blood pressure. **B**, **C** Boolean implication analysis used to identify cardiomyocyte- and fibroblast-specific gene sets. **D**, **E** Machine-learning analysis of RNA-seq data from CST-KO hearts identifies a 39-gene MyoFibro signature that is upregulated in untreated CST-KO mice and normalized following CST supplementation. **F**, **G** Composite MyoFibro score significantly distinguishes disease states (control vs HF) in human heart samples. **H** Composite MyoFibro score distinguishes disease states across publicly available datasets from human, mouse, and rat. Statistical significance was assessed using a two-sided Welch’s *t*-test. Predictive performance is reported as the receiver operating characteristic area under the curve (ROC–AUC). C control, CTRL control, CM cardiomyopathy, DCM diabetic cardiomyopathy, HF heart failure, SHR spontaneously hypertensive rat, WKY Wistar Kyoto. Graphical elements were created by the authors using Microsoft PowerPoint (Microsoft Consolidated UCSD Agreement) and NIH BioArt (https://bioart.niaid.nih.gov/). No generative AI tools were used in the creation of this figure.

Boolean analysis of RNA sequencing from CST-KO mouse hearts revealed upregulation of fibroblast and cardiomyocyte genes associated with HF that were reversed by CST supplementation. We have previously reported that CgA knockout (CgA-KO)^19^ and CST-KO^24^ mice are hypertensive. High blood pressure in CgA-KO and CST-KO mice was normalized after supplementation with CST (shown here in Fig. 1A as a schematic), implicating CST as an anti-hypertensive peptide^19,24^. In addition, we have shown that CST decreased infiltration of macrophages in the CST-KO heart and reduced fibrosis and inflammation^24^. Therefore, we undertook a transcriptomics approach to find out the loss and gain of function of left ventricular (LV) genes that are regulated by CST. We performed RNA sequencing from LVs of WT, CST-KO, and CST-KO mice supplemented with CST. The data were evaluated by Boolean analysis, which utilized a comprehensive dataset of 25,955 human samples (GSE119087) profiled using Affymetrix Human U133 Plus 2.0 microarrays (Fig. 1B). *SPARC* (Secreted Protein Acidic and Cysteine Rich), a seed gene known to be expressed in fibroblasts^28^ and myocytes^29^, was selected for the analysis. Boolean implication analysis [e.g., *SPARC* high → *TYROBP* (Transmembrane Immune Signaling Adaptor *TYROBP*) high] in blood and leukemia samples indicated that *SPARC* might also be expressed in some immune cell types (i.e., that express *TYROBP*). Therefore, we explored Boolean implications where *SPARC* low → X low to identify genes specific to fibroblasts and myocytes.

*COL6A3* (Collagen Type VI alpha 3 Chain) was identified as a candidate gene that is not expressed in immune cells but is highly expressed in fibroblasts and myocytes. Further analysis of Boolean equivalences involving *COL6A3* led to the identification of *DCN* (Decorin), which showed a Boolean equivalence relationship with *COL6A3* and was highly conserved between humans and mice. This relationship was exemplified by the robust Boolean implication *SPARC* low → *DCN* low. Based on these findings, we hypothesized that genes satisfying the Boolean implication *DCN* low → X low are specifically expressed in fibroblasts and myocytes (Fig. 1B).

We filtered genes that were characteristically upregulated in the LVs of untreated CST-KO mice and downregulated upon CST treatment. This analysis identified 39 genes constituting the “MyoFibro gene signature,” which are specific to fibroblasts and myocytes (Fig. 1C–E and S-Table 1). Composite score analysis revealed significant upregulation of the MyoFibro gene signature in untreated CST-KO mice (Fig. 1E) that largely corroborated with publicly available human HF patient tissue datasets (Fig. 1F, G). The composite score of the MyoFibro genes also distinguished disease state (control vs HF) in publicly available heart samples across human, mice, and rats (Fig. 1H).

### Boolean analysis identifies macrophage gene signatures dysregulated in CST-KO hearts

**Fig. 2:**
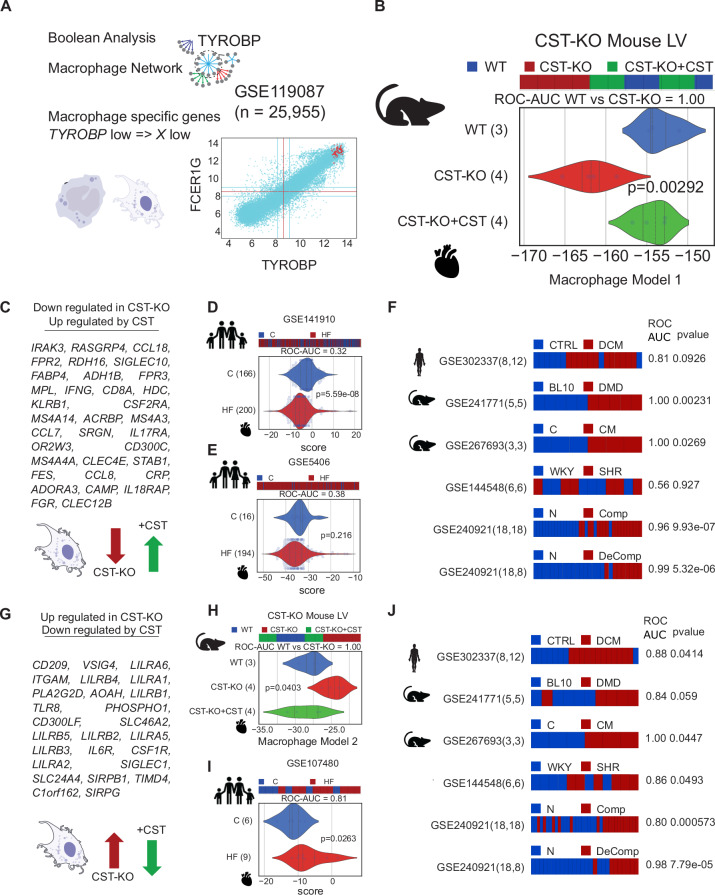
Boolean analysis of RNA-seq data identifies CST-regulated macrophage gene signatures. **A** Boolean implication analysis was used to identify macrophage-specific gene sets. **B**, **C** Machine-learning analysis of RNA-seq data from CST-KO hearts identifies a 34-gene Mac1 signature that is downregulated in untreated CST-KO mice and normalized following CST supplementation. **D**, **E** Composite Mac1 score significantly distinguishes disease states (control vs HF) in human heart samples. **F** The composite Mac1 score does not consistently distinguish disease states across publicly available datasets from human, mouse, and rat. (**G**, **H**) Machine-learning analysis identifies a 26-gene Mac2 signature that is upregulated in untreated CST-KO mice and normalized following CST supplementation. **I** The composite Mac2 score is significantly elevated in untreated CST-KO mice. **J** Composite Mac2 score significantly and consistently distinguishes disease states (control vs HF) across publicly available datasets from human, mouse, and rat. Statistical significance was assessed using a two-sided Welch’s *t*-test. Predictive performance is reported as the receiver operating characteristic area under the curve (ROC–AUC). Graphical elements were created by the authors using Microsoft PowerPoint (Microsoft Consolidated UCSD Agreement) and NIH BioArt (https://bioart.niaid.nih.gov/), including macrophage (IDs: 308, 311) and monocyte (ID: 356) illustrations. No generative AI tools were used in the creation of this figure.

Following a similar approach, we also identified macrophage-specific gene signatures (Macrophage Model 1 and Macrophage Model 2) using Boolean analysis (Fig. 2). Using the same dataset of 25,955 human samples (GSE119087), we performed Boolean implication analysis, which previously identified two universal macrophage genes, *TYROBP* and *FCER1G*^30^. We used the Boolean implication *TYROBP* low → X low to identify additional macrophage-specific genes (Fig. 2A). Genes that were downregulated in the LVs of untreated CST-KO mice and upregulated upon CST treatment were termed the “Mac1 gene signature”, comprising of 34 genes potentially expressed in various macrophage populations (Fig. 2B, C and S-Table 2). Composite score analysis revealed significant downregulation of the Mac1 gene signature in untreated CST-KO mice (Fig. 2B), with further validation showing significant downregulation in heart tissues of HF patients (Fig. 2D). In contrast, this signature was not significantly altered in a second HF dataset (GSE5406, Fig. 2E) and was upregulated in several additional human, mouse, and rat datasets (Fig. 2F).

Additionally, we identified a second macrophage-specific signature, called “Mac2”, comprising 26 genes that were upregulated in untreated CST-KO mice (Fig. 2G, H and S-Table 3). This Mac2 gene signature was significantly upregulated in the heart tissues of HF patients (Fig. 2I). While the Mac1 composite score did not consistently distinguish control from HF samples across additional public heart datasets from humans, mice, and rats, the Mac2 score reliably separated these disease states (Fig. 2F, J).

### CST restores insulin signaling and glucose metabolism in CST-KO hearts

**Fig. 3:**
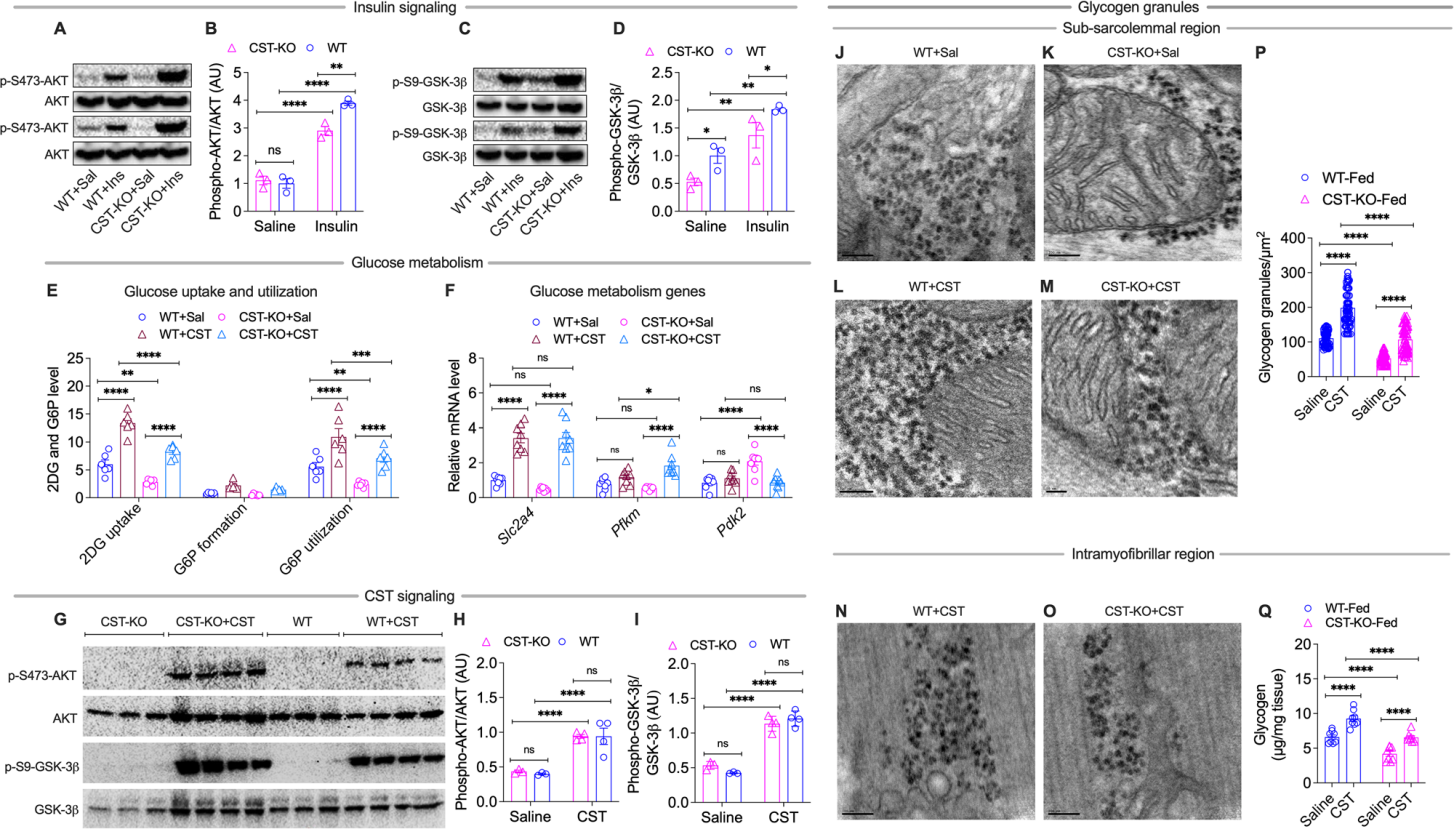
CST restores insulin signaling, glucose metabolism, and glycogen storage in the heart. **A** Representative immunoblots showing insulin-stimulated phosphorylation of AKT in wild-type (WT) and CST-KO hearts. **B** Densitometric quantification of AKT phosphorylation shown in (**A**) (*n* = 3). **C** Representative immunoblots showing insulin-stimulated phosphorylation of GSK-3β. **D** Densitometric quantification of GSK-3β phosphorylation shown in (**C**) (*n* = 3). **E** Glucose uptake and utilization in WT and CST-KO hearts in response to CST, including 2-deoxyglucose (2DG) uptake, glucose-6-phosphate (G6P) formation, and G6P utilization (*n* = 6). **F** Expression of genes involved in glucose metabolism in WT and CST-KO hearts in response to CST: *Slc2a4* (GLUT4, glucose transporter 4), *Pfkm* (phosphofructokinase, muscle type), and *Pdk2* (pyruvate dehydrogenase kinase 2). **G** Representative immunoblots showing CST regulation of AKT and GSK-3β phosphorylation. **H**, **I** Densitometric quantification of AKT (**H**) and GSK-3β (**I**) phosphorylation shown in (**G**) (*n* = 3–4). **J**–**M** Transmission electron micrographs of the subsarcolemmal region showing glycogen granules: saline-treated WT (**J**), saline-treated CST-KO (**K**), CST-treated WT (**L**), and CST-treated CST-KO (**M**) (*n* = 90 images). **N**, **O** Electron micrographs of the intermyofibrillar region showing glycogen granules in CST-treated WT (**N**) and CST-KO (**O**) hearts. **P** Morphometric quantification of glycogen granules. **Q** Biochemical determination of glycogen content in fed WT and CST-KO hearts (*n* = 8). Data are presented as mean ± SEM. Statistical analyses were performed using two-way ANOVA followed by Tukey’s multiple comparison test. *P* < 0.05 was considered significant. ^*^P < 0.05; ^**^P < 0.01; ^***^P < 0.001; ^****^P < 0.0001.

Transcriptomic analysis identified 99 (39 + 34 + 26) differentially expressed genes in CST-KO hearts associated with HF (Fig. 1D and 2C, G). Because metabolic dysregulation is a major driver of HF, we next focused on glucose utilization and insulin signaling pathways. Boolean network analysis revealed enrichment of *ITGAM*, *IFNG*, *ALDOB*, and *FPR2*, genes previously implicated in insulin resistance and altered glucose metabolism^31–34^. Consistent with this, insulin-stimulated phosphorylation of AKT (Ser473) and GSK-3β (Ser9) was reduced by ~33% and 25%, respectively, in CST-KO hearts compared to WT controls (Fig. 3A–D), indicating impaired insulin signaling.

To determine the functional impact, we measured glucose uptake and metabolism in isolated hearts. CST-KO hearts showed ~53% lower uptake of 2-deoxyglucose (2DG) and ~57% lower utilization of glucose-6-phosphate (G6P) than WT (Fig. 3E). CST supplementation reversed these defects, increasing glucose uptake 2.2-fold in WT and 2.9-fold in CST-KO hearts, and enhancing G6P utilization 1.95-fold in WT and 2.93-fold in CST-KO hearts (Fig. 3E). Because CST had no effect on G6P formation (Fig. 3E), the metabolic block appears downstream of glucose phosphorylation.

The rate-limiting glycolytic enzyme phosphofructokinase-1 (PFK-1), encoded by *Pfkm*, showed similar expression in WT and CST-KO hearts (Fig. 3F). However, CST increased *Pfkm* expression ~3.35-fold in CST-KO mice, consistent with enhanced glycolytic capacity.

In glucose oxidation, pyruvate dehydrogenase (PDH) catalyzes the conversion of pyruvate to acetyl-CoA, linking glycolysis to the Krebs cycle. PDH is inhibited by pyruvate dehydrogenase kinases (PDK1–4) and activated by pyruvate dehydrogenase phosphatases (PDP1/2)^35,36^. Since PDK2, the predominant cardiac isoform, is regulated by PPARs^37,38^, we assessed *Pdk2* expression. It was elevated ~2.45-fold in CST-KO hearts, suggesting reduced PDH activity and ATP production. CST treatment suppressed *Pdk2* by ~58%, consistent with restored OXPHOS and improved energy metabolism (Fig. 3F).

CST also enhanced AKT pathway activation. While basal phosphorylation of AKT (Ser473) and GSK-3β (Ser9) was comparable among saline-treated groups, CST significantly increased AKT phosphorylation (~2.19-fold in CST-KO; ~2.35-fold in WT) and GSK-3β phosphorylation (~2.13-fold in CST-KO; ~2.81-fold in WT) (Fig. 3G–I). These results confirm that CST restores insulin sensitivity in CST-deficient hearts.

Although cardiac glycogen stores are limited^39^, they are crucial for oxygen-efficient energy supply under stress^40^. In the sub-sarcolemmal region, glycogen granules were reduced by ~54% in saline-treated CST-KO hearts relative to WT (Fig. 3J, K, P). CST treatment increased glycogen content 2.12-fold in CST-KO and 1.64-fold in WT hearts, respectively (Fig. 3L, M, P). Consistent with the sub-sarcolemmal region, glycogen granules in the intermyofibrillar region were also reduced by ~36% in saline-treated CST-KO hearts relative to WT (Fig. 3N, Q). CST also increased glycogen content ~1.6-fold in CST-KO and 1.4-fold in WT hearts (Fig. 3O, Q), respectively. Thus, CST promotes glycogen synthesis and storage alongside improved insulin signaling.

### CST improves systemic glucose homeostasis and insulin sensitivity

**Fig. 4:**
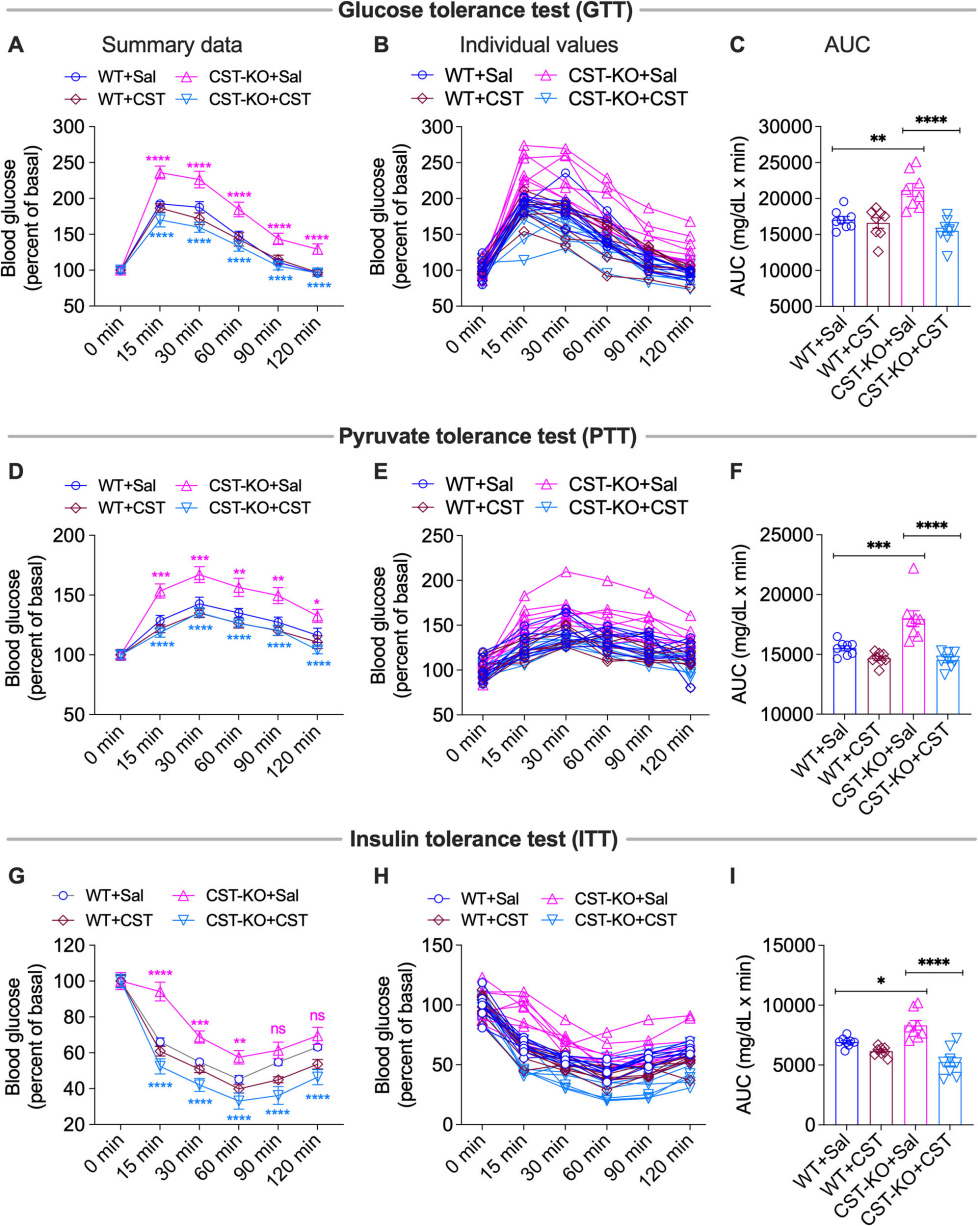
CST improves systemic glucose homeostasis, suppresses hepatic glucose output, and enhances insulin sensitivity. **A** GTT (2 g/kg dextrose, intraperitoneal) showing mean blood glucose curves for saline-treated WT and CST-KO mice and CST-treated WT and CST-KO mice (*n* = 8 per group). **B** Individual GTT blood glucose traces from the same cohorts (*n* = 8). **C** Quantification of GTT area under the curve (AUC; mg/dL·min) (*n* = 8). **D** PTT (2 g/kg sodium pyruvate, intraperitoneal) showing mean blood glucose curves for saline-treated WT and CST-KO mice and CST-treated WT and CST-KO mice (*n* = 8 per group). **E** Individual PTT blood glucose traces (*n* = 8). **F** Quantification of PTT AUC (mg/dL·min) (*n* = 8). **G** ITT (0.5 IU/kg human insulin, intraperitoneal) showing mean blood glucose curves for saline-treated WT and CST-KO mice and CST-treated WT and CST-KO mice (*n* = 8 per group). **H** Individual ITT blood glucose traces (*n* = 8). **I** Quantification of ITT AUC (mg/dL·min) (*n* = 8). Data are presented as mean ± SEM. Statistical analyses were performed using one- or two-way ANOVA followed by Tukey’s multiple comparison test. *P* < 0.05 was considered significant. ^**^P < 0.01; ^***^P < 0.001; ^****^P < 0.0001.

To evaluate systemic metabolic effects, we performed glucose, pyruvate, and insulin tolerance tests (ITTs). In glucose tolerance tests (GTT), CST-treated CST-KO mice showed improved glucose excursions (Fig. 4A–C). In pyruvate tolerance tests (PTT), CST reduced glucose output (Fig. 4D–F), indicating suppressed gluconeogenesis. In ITT, CST induced pronounced hypoglycemia in CST-KO mice (Fig. 4G–I). Collectively, these findings establish CST as a glucose-lowering, insulin-sensitizing peptide that reverses metabolic inflexibility in the CST-KO heart.

### CST reverses lipid-induced insulin resistance and suppresses fatty acid metabolism

**Fig. 5:**
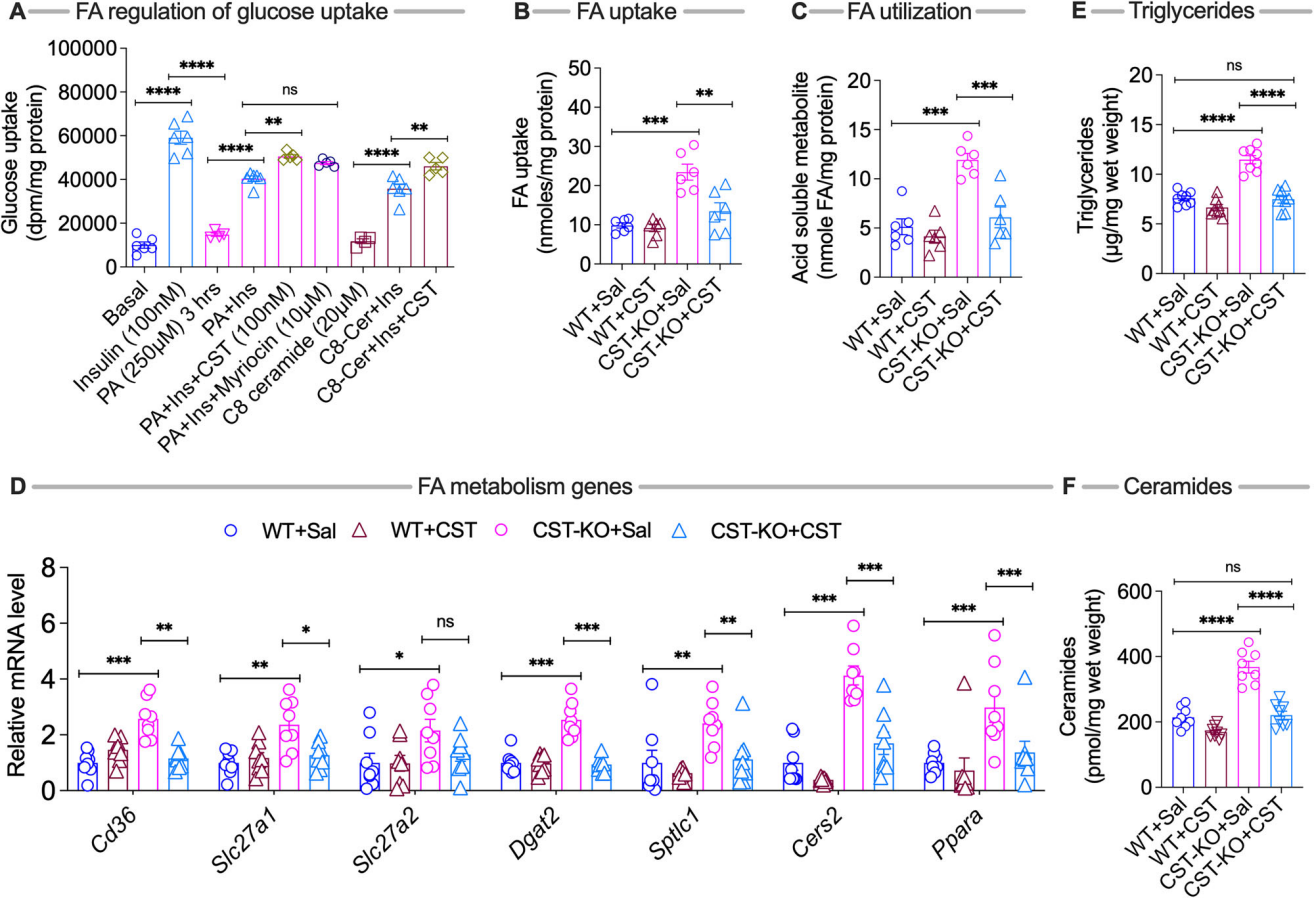
CST suppresses fatty acid uptake, oxidation, and lipotoxicity in the heart. **A** Modulation of insulin-stimulated glucose uptake by FA in WT cardiomyocytes (*n* = 4–8). **B** FA uptake in WT and CST-KO hearts in response to CST (*n* = 6). **C** FA utilization (β-oxidation) in WT and CST-KO hearts in response to CST (*n* = 6). **D** Expression of genes involved in lipid metabolism in WT and CST-KO hearts in response to CST (*n* = 8): *Cd36* (fatty acid translocase, FAT); *Slc27a1* (fatty acid transport protein 1, FATP1); *Slc27a2* (fatty acid transport protein 2, FATP2); *Dgat2* (diacylglycerol O-acyltransferase 2); *Sptlc1* (serine palmitoyltransferase long-chain base subunit 1); *Cers2* (ceramide synthase 2); and *Ppara* (peroxisome proliferator-activated receptor-α). **E** Triglyceride content in WT and CST-KO hearts in response to CST (*n* = 8). **F** Ceramide content in WT and CST-KO hearts in response to CST (*n* = 8). Data are presented as mean ± SEM. Statistical analyses were performed using one- or two-way ANOVA as appropriate, followed by Tukey’s multiple comparison test. *P* < 0.05 was considered significant. ^*^P < 0.05; ^**^P < 0.01; ^***^P < 0.001; ^****^P < 0.0001.

Activation of insulin signaling promotes GLUT4 translocation to the sarcolemma, thereby enhancing cardiomyocyte glucose uptake. In cardiomyocytes isolated from lean C57BL/6 (WT) mice, insulin stimulation elicited a ~5.9-fold increase in glucose uptake (Fig. 5A). Co-incubation with palmitic acid (PA) reduced insulin-stimulated glucose uptake by ~32%, indicating PA-induced insulin resistance (Fig. 5A). Remarkably, inclusion of CST in the incubation medium not only restored insulin responsiveness but further enhanced glucose uptake by ~1.25-fold relative to insulin alone (Fig. 5A). Similarly, co-incubation with ceramide decreased insulin-stimulated glucose uptake by ~39%, underscoring the combined lipotoxic effects of PA and ceramide on insulin sensitivity^41–43^. CST treatment effectively counteracted this inhibition, augmenting glucose uptake by ~1.29-fold (Fig. 5A), demonstrating that CST mitigates lipid-induced insulin resistance and enhances myocardial insulin sensitivity.

FA uptake was markedly elevated (~2.4-fold) in CST-KO hearts compared with WT (Fig. 5B). CST supplementation decreased FA uptake by ~43% in CST-KO hearts (Fig. 5B). In cardiac muscle, mitochondrial FA β-oxidation generates acetyl-CoA to fuel the Krebs cycle and ATP synthesis. Consistent with enhanced FA oxidation, acid-soluble metabolites (ASM; containing acetyl-CoA) were ~2.3-fold higher in CST-KO than WT hearts (Fig. 5C). CST treatment reduced FA β-oxidation by ~49% (Fig. 5C), indicating a metabolic shift away from ATP generation through FA oxidation.

Next, we analyzed the expression of genes associated with FA metabolism and insulin resistance. Boolean network analysis identified upregulation of *ITGAM, IFNG, ALDOB, FPR2, MS4A4A*, and *PLA2G2D* - all implicated in lipid metabolism and insulin resistance^32–34,44–46^. Although passive diffusion contributes modestly, myocardial FA uptake predominantly depends on transporters such as CD36, plasma membrane fatty acid–binding protein (FABPpm), and fatty acid transport proteins FATP1 and FATP2 (encoded by *Slc27a1* and *Slc27a2*)^47^. Consistent with elevated FA influx, CST-KO hearts exhibited higher transcript levels of *Cd36* (~2.6-fold), *Slc27a1* (~2.4-fold), and *Slc27a2* (~2.2-fold; Fig. 5D). CST treatment decreased *Cd36* (~55%) and *Slc27a1* (~46%) expression, concordant with its suppression of FA uptake (Fig. 5D).

Excess intracellular FA is normally sequestered into triglycerides (TGs) for safe storage in lipid droplets^48,49^, with TG biosynthesis catalyzed by diacylglycerol acyltransferase 2 (DGAT2). CST-KO hearts displayed ~2.5-fold higher *Dgat2* expression, consistent with increased TG synthesis (Fig. 5E). Moreover, CST-KO hearts showed strong upregulation of ceramide biosynthetic genes, including *Sptlc1* (~2.4-fold) and *Cers2* (~4.1-fold) (Fig. 5D), leading to elevated ceramide content (Fig. 5F). Long-chain ceramides are known mediators of insulin resistance, mitochondrial dysfunction, and cardiac fibrosis^48,50^. CST supplementation markedly reduced *Sptlc1* (~54%) and *Cers2* (~59%) expression (Fig. 5D) and correspondingly decreased myocardial ceramide content (Fig. 5F).

Finally, *Ppara*, a master transcriptional regulator of lipid metabolism^51,52^, was upregulated ~3-fold in CST-KO hearts (Fig. 5D). CST treatment reduced *Ppara* expression by ~54%, paralleling its inhibitory effects on FA transport and oxidation genes. Together, these findings demonstrate that CST restores metabolic balance by suppressing excessive FA uptake, oxidation, and ceramide biosynthesis - thereby preventing maladaptive lipid accumulation and preserving metabolic flexibility in CST-KO hearts.

### CST restores sarcomeric structure and mitochondrial ultrastructure in CST-KO hearts

Transcriptomic analyses revealed dysregulation of genes linked to cardiac contractility and mitochondrial integrity, prompting an ultrastructural evaluation of myofibrils and mitochondria. LV tissue was collected during diastole following perfusion with cardioplegic solution to preserve native morphology.

**Fig. 6:**
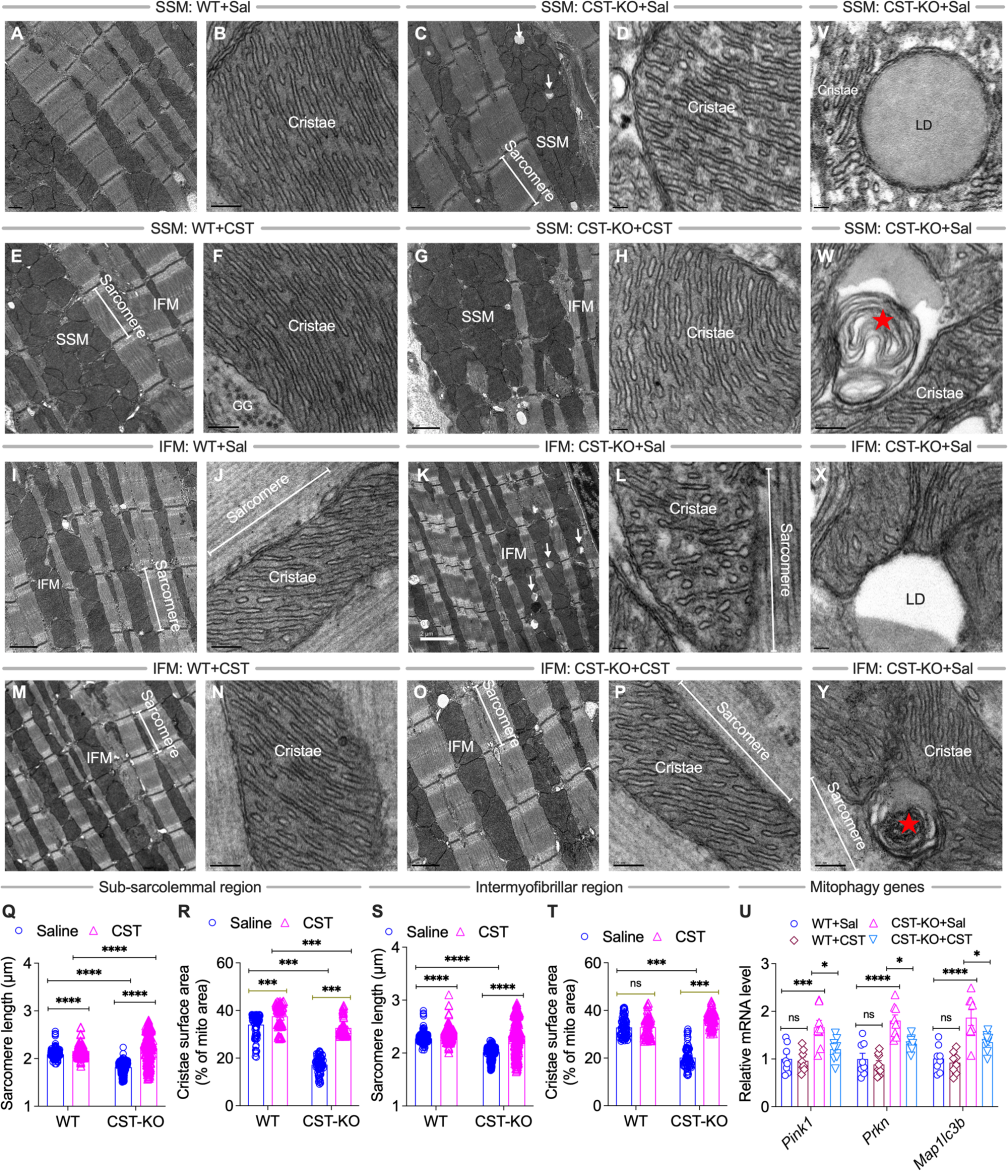
CST restores sarcomeric organization, mitochondrial ultrastructure, and mitophagy in the heart. Subsarcolemmal mitochondria (SSM). **A**, **C**, **E**, **G** Low-magnification electron micrographs showing sarcomeres and mitochondria in saline-treated WT (**A**), saline-treated CST-KO (**C**), CST-treated WT (**E**), and CST-treated CST-KO (**G**) hearts. **B**, **D**, **F**, **H** High-magnification images of SSM showing mitochondrial cristae in the corresponding groups. Inter-/intramyofibrillar mitochondria: (IFM): **I**, **K**, **M**, **O** Low-magnification electron micrographs showing sarcomeres and mitochondria in saline-treated WT (**I**), saline-treated CST-KO (**K**), CST-treated WT (**M**), and CST-treated CST-KO (**O**) hearts. **J**, **L**, **N**, **P** High-magnification images of IFM showing mitochondrial cristae in the corresponding groups. **Q** Morphometric analysis of sarcomere length in the subsarcolemmal region (*n* = 265–303). **R** Quantification of SSM cristae surface area (*n* = 84–85). **S** Morphometric analysis of sarcomere length in the inter-/intramyofibrillar region (*n* = 265–303). **T** Quantification of IFM cristae surface area (*n* = 84–85). **U** Expression of mitophagy-associated genes (*Pink1, Prkn*, and *Map1lc3b*) in saline- and CST-treated WT and CST-KO hearts (*n* = 8). **V**, **X** High-magnification images showing lipid droplets in SSM (**V**) and IFM (**X**). **W**, **Y** High-magnification images showing mitophagy in SSM (**W**) and IFM (**Y**). SSM sub-sarcolemmal mitochondria, IFM inter-/intramyofibrillar mitochondria; Lipid droplets are indicated by small arrows, and mitophagy by asterisks. Data are presented as mean ± SEM. Statistical analyses were performed using two-way ANOVA followed by Tukey’s multiple comparison test. *P* < 0.05 was considered significant. ^*^P < 0.05; ^***^P < 0.001; ^****^P < 0.0001.

CST-KO hearts exhibited conspicuous sarcomeric disorganization, with shortened sarcomeres in both subsarcolemmal (SSM; ~13% reduction) and intermyofibrillar (IFM; ~11% reduction) regions compared with WT controls (Fig. 6A–D, I–L, Q, S). CST supplementation rescued these structural defects, increasing sarcomere length by ~22% in SSM and ~15% in IFM regions, whereas WT hearts showed only modest (~3%) elongation upon CST treatment (Fig. 6E–H, M–P, Q, S).

Mitochondrial ultrastructure was also severely perturbed in CST-KO cardiomyocytes. In the SSM region, cristae surface area was reduced by ~49% relative to WT (Fig. 6B, D, R), while CST treatment restored cristae density, producing an ~89% increase compared with WT controls (~10% increase; Fig. 6F, H, R). Similarly, IFM mitochondria from CST-KO hearts displayed a ~ 39% reduction in cristae surface area, which was rescued by CST supplementation (~81% increase vs <1% in WT; Fig. 6J, L, N, P, T).

Consistent with excessive lipid accumulation, CST-KO cardiomyocytes contained abundant lipid droplets in both SSM (Fig. 6C, V) and IFM (Fig. 6K, X) regions. Because lipid overload is known to trigger mitophagy^49^, we also observed mitophagic vesicles in CST-KO hearts (SSM: Fig. 6W; IFM: Fig. 6Y), but not in WT controls.

### CST regulates mitophagy and mitochondrial quality control pathways

To determine whether CST regulates mitochondrial quality control programs in the heart, we analyzed mitophagy markers using bulk RNA sequencing followed by qRT-PCR validation. RNA-seq revealed a robust enrichment of the “positive regulation of mitophagy” gene set in CST-KO mice, with prominent upregulation of *PRKN* (*Park2*) and a significant elevation in the mitophagy gene-set score relative to WT (S-Fig. 1 and S-Table 4). CST supplementation reversed these transcriptomic changes, reducing both PRKN pathway activity and the global mitophagy score (S-Fig. 1 and S-Table 4). Complementary RT-qPCR analyses further confirmed these findings: *Pink1*, *PRKN* (aka *Park2)*, and *Map1lc3b* (aka *LC3B*) mRNA levels were all increased in CST-KO hearts and restored to near WT levels with CST treatment (Fig. 6U). Together, these parallel transcriptomic and molecular assays demonstrate that CST deficiency activates the PINK1 → Parkin → LC3 mitophagy axis, whereas CST supplementation restores mitochondrial homeostasis.

### CST regulation of sarcomeric and mitochondrial genes

Transcriptomic analysis revealed broad downregulation of sarcomeric gene networks in CST-KO hearts, prompting validation by qRT–PCR. Consistent with Boolean network analysis, expression of *Actc1* (α-cardiac actin) was markedly reduced (~72%) in CST-KO hearts. Several other sarcomeric components were also significantly downregulated, including *Tnni3* (Troponin I3; ~66%), *Tnnt2* (Troponin T2; ~65%), *Myl4* (Myosin light chain 4; ~70%), *Myl7* (Myosin light chain 7; ~70%), and *Myh7* (Myosin heavy chain 7; ~70%) (S-Fig. 2A). CST supplementation restored the expression of these sarcomeric genes to near-WT levels (S-Fig. 2A). In contrast, *Tpm1* and *Ttn* expression remained comparable between WT and CST-KO hearts (S-Fig. 2A), indicating selective regulation of contractile gene subsets by CST.

Given the mitochondrial structural abnormalities observed in CST-KO cardiomyocytes, we next evaluated mitochondrial gene expression. qRT–PCR revealed pronounced reductions in *Ndufa3* (NADH: ubiquinone oxidoreductase subunit A3; ~77%), *Sdhc* (succinate dehydrogenase complex subunit C; ~76%), and *Atp5pf* (ATP-synthase peripheral stalk subunit F6; ~76%) (S-Fig. 2B). CST treatment robustly enhanced the expression of these genes, increasing *Ndufa3* (~15-fold), *Sdhc* (~13-fold), and *Atp5pf* (~13-fold) (S-Fig. 2B). Together, these findings indicate that CST not only preserves sarcomeric integrity but also restores mitochondrial respiratory gene expression, thereby reinforcing the structural and energetic homeostasis of the myocardium.

### CST directly interacts with mitochondrial ATP synthase

**Fig. 7:**
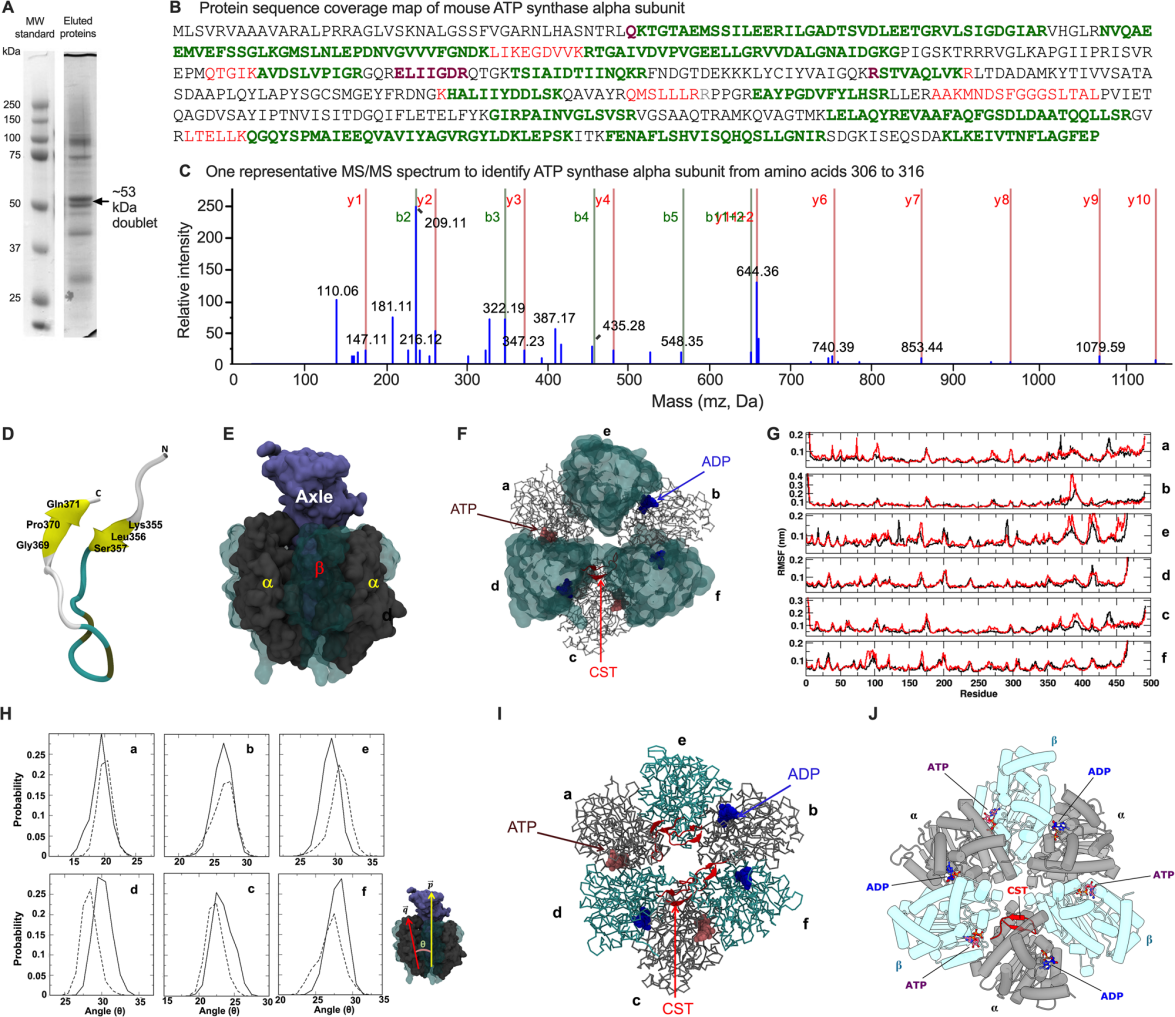
CST binds mitochondrial F₁-ATP synthase and modulates its structural dynamics. **A** SDS–PAGE analysis of proteins eluted from biotinylated CST–streptavidin agarose following ligand affinity chromatography of mouse heart homogenate using human CST (hCgA352–372) as bait. Bound proteins were eluted at pH 5.5 and analyzed. **B** Sequence coverage map of mouse ATP synthase α-subunit identified by trypsin–LC–MS/MS from CST-binding proteins. Peptide confidence is indicated as follows: green, ≥95%; purple, ≥50%; red, lower confidence. **C** Representative MS/MS spectrum (precursor mass 1286.7) of a peptide derived from the CST-binding protein. **D** Time-averaged structure of CST shown in cartoon representation, with N- and C-termini and selected residues indicated. **E** Structure of the F₁-ATP synthase complex, with α-subunits shown in gray, β-subunits in cyan, and the central axle in blue. **F** Molecular docking of CST to F₁-ATP synthase. α-subunits (chains a–c) are shown in gray and β-subunits (chains d–f) in cyan. ATP and ADP molecules are shown in magenta and blue, respectively. CST (red) binds at the α–β interface corresponding to the lowest-energy complex. The central axle is omitted for clarity. **G** Root mean square fluctuation (RMSF) analysis of F₁-ATP synthase residues comparing apo (red) and CST-bound (black) states. **H** Probability distribution of subunit angular displacement relative to the central axle for apo (solid lines) and CST-bound (dotted lines) F₁-ATP synthase. **I** Progressive docking of multiple CST molecules onto F₁-ATP synthase, showing binding at distinct α–β interfaces. **J** Structural model of the CST–F₁-ATP synthase complex predicted by AlphaFold3. The α-subunit is shown in gray, the β-subunit in cyan, and CST in red. Bound nucleotides are shown in licorice representation (ADP, blue; ATP, magenta). The γ-subunit is omitted for clarity.

Given CST’s role in regulating cardiac bioenergetics, we sought to identify its molecular binding partners. Using ligand affinity chromatography, murine heart homogenates were incubated with biotinylated human CST immobilized on a streptavidin column. CST-bound proteins were separated by gel electrophoresis, and coomassie staining revealed distinct protein bands at approximately 100, 53, 43, and 30 kDa (Fig. 7A). Tryptic digestion followed by LC–MS/MS identified the intense 53 kDa doublet as the α- and β-subunits of the F₁-ATP synthase complex (Fig. 7B, C and S-Fig. 3), with the β-subunit showing 74% sequence coverage (106 spectra, rank 1; S-Table 5) and the α-subunit 58% coverage (79 spectra, rank 2; S-Table 5). These results indicate that CST interacts with cardiac ATP synthase. Minor associations were also detected with ER Ca²⁺-binding proteins (sarcalumenin, calsequestrin) and common cytoskeletal contaminants (tubulin, keratin; S-Table 5). Peptide Summary is shown in Supplementary Data 1.

### Computational and structural analyses support CST binding to ATP synthase

Computational modeling further supported this interaction. A 250 ns molecular dynamics (MD) simulation revealed that CST adopts a metastable antiparallel β-sheet conformation with transient transitions to coil and 3₁₀-helix states (Fig. 7D). In its β-sheet form, the N-terminal strand was stabilized by Lys355–Ser357 and the C-terminal strand by Gly369–Gln371. The modeled murine F₁-ATP synthase structure resembled the bovine crystal structure^53^, composed of alternating α- and β-subunits arranged around a central γ–δ–ε axle (Fig. 7E). LC–MS/MS and time-of-flight (TOF) data suggested CST binds at the α–β interface and docking localized the peptide near the d–c–f interface (Fig. 7F). Structural analysis revealed hydrogen bonding between CST and Glu341/Tyr381 (D-subunit), as well as Ser382/Asp315 (F-subunit), accompanied by extensive hydrophobic interactions across the c, d, and f subunits (S-Fig. 4). Notably, CST residues Ser352, Arg361, Arg366, and Pro370 bridged adjacent subunits, suggesting stabilization of the α–β interface.

Comparative MD simulations of CST-bound vs unbound ATP synthase showed reduced atomic fluctuations at the d–c–f interface and increased flexibility at the opposite a–e–b interface (Fig. 7G). Angular displacement analysis confirmed decreased tilt of d–c–f subunits and increased tilt of a–e–b subunits relative to the central axle (Fig. 7H). These conformational shifts are consistent with prior models proposing that alternating subunit displacements enable ADP binding and ATP release^54^. Progressive docking of multiple CST copies to F1-ATPase was performed to assess whether CST can bind additional α–β interfaces. Notably, the results indicate that up to four CST peptides can be accommodated within the receptor, each engaging a distinct α–β interface. The predicted binding pattern consistently implicated residues Ser352, Ser353, Arg361, Arg366, Pro370, Gln371, and Leu372 across the four interfaces (Fig. 7I and S-Fig. 5).

To independently validate CST binding to F₁-ATP synthase, we performed AlphaFold3 multimer modeling incorporating the α-, β-, and γ-subunit sequences together with the CST peptide. Models were ranked by interchain predicted TM-score (ipTM) and predicted TM-score (pTM), with the top-ranked model (ipTM = 0.82; pTM = 0.84) selected for detailed analysis owing to its high confidence and well-defined interaction interface (Fig. 7J). Remarkably, the AlphaFold-predicted CST binding site overlapped with the α–β interface identified by our docking and MD simulations. The model also reproduced the transient antiparallel β-sheet conformation of CST observed in simulations, underscoring the peptide’s structural adaptability during binding.

Importantly, inclusion of three ATP and three ADP molecules in the AlphaFold model positioned these nucleotides precisely within the catalytic sites, confirming the physiological plausibility of the CST–ATP synthase complex. The strong concordance between AlphaFold predictions, MD simulations, and biochemical evidence provides compelling validation that CST interacts directly with the α–β interface of F₁-ATP synthase to modulate its catalytic dynamics.

### CST enhances mitochondrial bioenergetics in cardiomyocytes

**Fig. 8:**
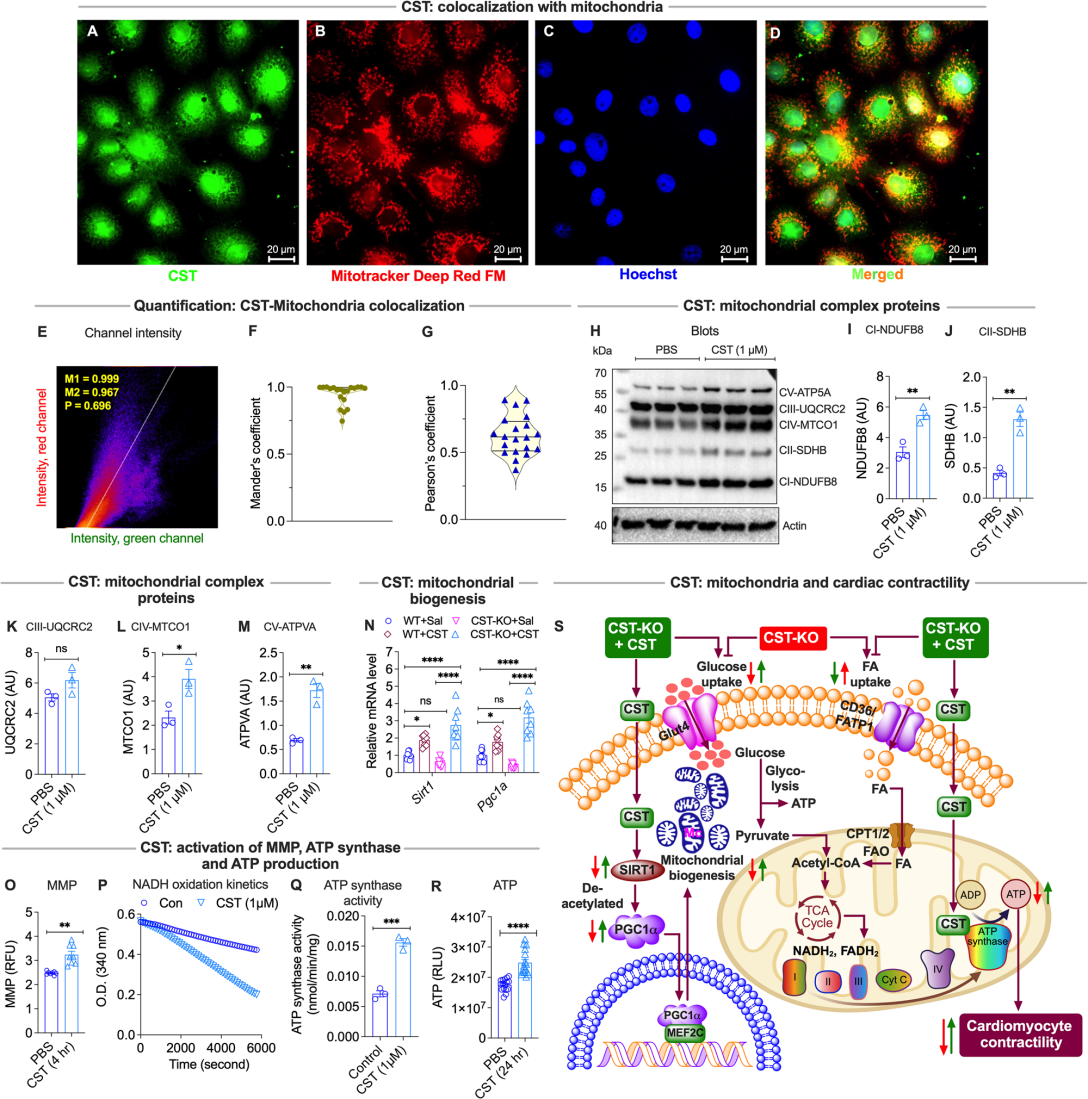
CST localizes to mitochondria and enhances mitochondrial bioenergetics in cardiomyocytes. **A**–**D** Fluorescence microscopy of H9c2 cells showing co-localization of endogenous CST with mitochondria: CST (**A**; AF488), mitochondria (**B**; MitoTracker™ Deep Red FM), nuclei (**C**; Hoechst), and merged image (**D**), in which co-localization appears yellow. Images were acquired using a Keyence microscope. Scale bar, 20 µm. **E** Representative intensity profiles of red (mitochondria) and green (CST) channels (*n* = 20 cells). M1 and M2 denote Manders’ coefficients (fractional overlap of red with green and green with red channels, respectively), and P denotes Pearson’s correlation coefficient. **F**, **G** Scatter plots showing the distribution of Manders’ coefficients (**F**) and Pearson’s correlation coefficients (**G**) across individual cells (*n* = 20). **H** Representative immunoblots showing expression of mitochondrial OXPHOS complex proteins. **I**–**M** Densitometric quantification of OXPHOS subunits: Complex I (Ndufb8), Complex II (Sdhb), Complex III (Uqcrc2), Complex IV (Mt-co1), and Complex V (Atp5a) (*n* = 3). (N) Relative mRNA expression of Sirt1 and Pgc1a (*n* = 8). **O** Mitochondrial membrane potential (ΔΨm) (*n* = 8). **P** Time-course analysis of ATP synthase (Complex V) activity in mitochondria isolated from control and CST-treated (1 µM, 24 h) H9c2 cells (*n* = 3). Enzymatic activity was measured by monitoring NADH oxidation at 340 nm. **Q** Quantification of Complex V activity normalized to protein concentration (nmol·min⁻¹·mg⁻¹) (*n* = 3). **R** Intracellular ATP levels in H9c2 cells following CST treatment (1 µM, 24 h) (*n* = 16). **S** Schematic illustrating the proposed mechanism of CST action on cardiac metabolism and mitochondrial function. CD36 fatty acid translocase, CPT1/2 carnitine palmitoyltransferases 1 and 2, CST catestatin, Cyt c cytochrome c, FA fatty acid, FAO fatty acid oxidation, FADH₂ flavin adenine dinucleotide, FATP1 fatty acid transport protein 1, GLUT4 glucose transporter 4, Mito mitochondria, NADH nicotinamide adenine dinucleotide, PGC1α peroxisome proliferator-activated receptor gamma coactivator 1-alpha, SIRT1 sirtuin 1. Data are presented as mean ± SEM. Statistical significance was assessed using a two-sided *t*-test or two-way ANOVA, as appropriate, followed by Tukey’s multiple comparison test. A value of *P* < 0.05 was considered significant. ^*^P < 0.05; ^**^P < 0.01; ^***^P < 0.001; ^****^P < 0.0001. **S** Created by the authors using ChemDraw 20.1 (UC San Diego institutional license), BioRender (Jati, S., 2026; https://BioRender.com/1q2s9wr), and Affinity software. No generative AI tools were used in the creation of this figure.

CST has previously been reported to enter diverse cell types, including neutrophils, macrophages, bacteria, and fungi^55,56^. We used FITC-conjugated CST to discern penetration of CST into neonatal cardiomyocytes. Primary cardiomyocytes from C57BL/6 WT mice were cultured and treated with FITC-CST. It was observed that CST could enter the cardiomyocytes and co-localized on the mitochondria of the cardiomyocytes that were stained with MitoTracker® Deep Red FM (S-Fig. 6A–F). To refine its subcellular distribution, we also detected endogenous CST by immunofluorescence using an AF488-conjugated secondary antibody (green), and mitochondria labeled with MitoTracker® Deep Red FM (red) in H9c2 cells. Nuclei were counterstained with Hoechst. Merged images revealed extensive co-localization of CST with mitochondria (Fig. 8A–D). Quantitative analysis further confirmed this enrichment: intensity profiling showed synchronous CST-mitochondria signal (Fig. 8E). Mander’s coefficient demonstrated that 99.9% of mitochondria signal overlapped with CST signal (M1: Fig. 8E, F) and 96.7% of CST signal overlapped with mitochondria (M2: Fig. 8E). Further, Pearson’s correlation coefficient indicated a strong positive association between the two channels (*P* = 0.696: Fig. 8E, G). Together, these data indicate that CST localizes predominantly to mitochondria in cardiomyocytes.

To determine whether mitochondrial localization translates to functional effects, we next examined CST-mediated regulation of OXPHOS. CST increased the abundance of several OXPHOS complex subunits, including complex I NDUFB8 (~1.79-fold), complex II SDHB (~3.17-fold), complex IV MTCO1 (~1.68-fold), and complex V ATP5A (~2.49-fold), whereas levels of the complex III subunit UQCRC2 remained unchanged (Fig. 8H–M). Consistently, CST upregulated the expression of mitochondrial biogenesis regulators *Sirt1* and *Pgc1a* (Fig. 8N).

To determine whether CST enhances mitochondrial function, we assessed mitochondrial membrane potential (Δψm) in neonatal mouse cardiomyocytes, ATP synthase (Complex V) activity in H9c2 cardiomyocytes, and total cellular ATP levels in neonatal mouse cardiomyocytes. CST treatment significantly increased mitochondrial membrane potential compared with PBS-treated controls (Fig. 8O), indicating enhanced electrochemical gradient integrity. Consistent with this observation, mitochondria isolated from CST-treated cells exhibited increased ATP synthase catalytic activity. Kinetic analysis demonstrated a greater rate of NADH oxidation in CST-treated mitochondria (Fig. 8P), and quantitative analysis confirmed a significant elevation in Complex V activity (Fig. 8Q). Importantly, the enhancement in mitochondrial enzymatic function translated into increased intracellular ATP levels following 24 h CST treatment (Fig. 8R). Together, these findings demonstrate that CST augments mitochondrial bioenergetic capacity by strengthening membrane potential, increasing ATP synthase activity, and elevating ATP production in cardiomyocytes.

To further evaluate CST’s impact on mitochondrial respiration, we conducted Seahorse XF Mito Stress Tests in H9c2 cells. CST did not significantly alter basal oxygen consumption (S-Fig. 7A), non-mitochondrial respiration (S-Fig. 7B), basal respiration (S-Fig. 7C), proton leak (S-Fig. 7D), ATP-linked respiration (S-Fig. 7E), maximal respiration (S-Fig. 7F), or spare respiratory capacity (S-Fig. 7G). Seahorse ATP Rate assays similarly revealed no CST-dependent changes in glycolytic, mitochondrial, or total ATP production (S-Fig. 7H–J).

Although CST markedly increased Δψm (Fig. 8O) and ATP content (Fig. 8P) in neonatal primary cardiomyocytes, these effects were not recapitulated in H9c2 cells. This divergence is consistent with established metabolic differences between the two models. Neonatal cardiomyocytes possess a well-developed mitochondrial network, depend predominantly on OXPHOS, and exhibit strong spare respiratory capacity - conditions that allow CST to enhance Δψm and ATP synthesis.

By contrast, H9c2 myoblast-derived cells rely primarily on glycolysis and display limited mitochondrial oxidative capacity, particularly when maintained in standard high-glucose media. Prior studies have shown that high-glucose H9c2 cells exhibit markedly reduced oxygen consumption, low respiratory control ratios, and increased lactate generation, confirming a predominantly glycolytic phenotype^57^. Additional work demonstrates that undifferentiated H9c2 cells are metabolically immature and shift toward oxidative metabolism only upon differentiation^58^. Moreover, comprehensive transcriptomic and functional profiling reveals that cardiac cell lines such as H9c2 have restricted metabolic responsiveness compared with primary cardiomyocytes^59^. These metabolic constraints likely explain why CST-dependent increases in OCR or ATP production were not observed in H9c2 cells. Thus, CST’s bioenergetic effects are model-dependent, with robust activity in metabolically competent primary cardiomyocytes but minimal impact in glycolytic, low-reserve H9c2 cells. A schematic summarizing our proposed mechanism is shown in Fig. 8S.

## Discussion

This study identifies catestatin (CST) as a key regulator of cardiac metabolic and structural homeostasis, linking its endocrine functions to mitochondrial control of energy metabolism. Through integrated transcriptomic, biochemical, ultrastructural, and computational analyses, we show that CST reverses lipid-induced insulin resistance, normalizes fatty acid utilization, restores mitochondrial architecture, and enhances myocardial ATP production. Importantly, direct measurements demonstrate that CST increases ATP synthase (Complex V) activity in cardiac mitochondria, accompanied by elevated mitochondrial membrane potential (ΔΨm) and intracellular ATP levels. Structural modeling and biochemical data support an interaction between CST and the F₁-ATP synthase complex at the α–β catalytic interface, potentially facilitating conformational transitions required for efficient catalysis. These findings extend CST from a circulating cardioprotective peptide to a direct modulator of mitochondrial bioenergetics, highlighting its therapeutic relevance in HF and metabolic cardiomyopathy.

CST is increasingly recognized as a pleiotropic regulator of cardiovascular function, acting through multiple conserved mechanisms. It lowers blood pressure in monogenic^19,24,60^ and polygenic^20,21,61^ models of hypertension and in humans^62^; modulates inotropy and lusitropy across vertebrate species^63–65^; promotes pre- and post-conditioning-induced cardioprotection^24,66^; and preserves baroreflex sensitivity^67^ and heart rate variability^68,69^. The present work adds a new dimension to CST biology—its ability to restore cardiac metabolic flexibility in insulin-resistant and lipotoxic states, coupled to enhanced ATP synthase activity, increased ΔΨm, and augmented ATP production.

Under physiological conditions, the heart maintains function through balanced oxidation of FA and glucose^10,70^, with substrate preference adapting dynamically to metabolic demand^7,71,72^. In disease states, this balance is disrupted. CST deficiency produces a diabetic-like phenotype characterized by excessive FA oxidation and reduced glucose utilization. Because FA oxidation requires greater oxygen consumption per ATP generated^73^, this shift imposes energetic inefficiency and contributes to metabolic inflexibility. The resulting bioenergetic stress likely contributes to the previously reported loss of ischemic preconditioning in CST-KO mice^24^. Enhancement of ATP synthase activity by CST provides a mechanistic basis for restoration of energetic efficiency.

At the signaling level, impaired insulin-stimulated phosphorylation of AKT and GSK-3β in CST-KO hearts reflects disrupted glucose metabolism. Excess FA uptake and incomplete β-oxidation increase NADH^74^, acetyl-CoA, and citrate, which inhibit PDH and PFK-1^75,76^, thereby suppressing glucose oxidation and GLUT4-mediated glucose uptake. Elevated acetyl-CoA further impairs pyruvate oxidation, favoring lactate accumulation and intracellular acidosis^12,77^, a known contributor to contractile dysfunction^78^. Upregulation of ceramide biosynthesis further exacerbates insulin resistance and mitochondrial dysfunction. By enhancing ΔΨm and ATP synthase activity, CST interrupts this maladaptive metabolic cycle and restores bioenergetic balance.

CST deficiency also disrupts sarcomeric integrity. Expression of key contractile genes—including *Tnni3*, *Tnnt2*, *Actc1*, *Myl4*, *Myl7*, and *Myh7*—was markedly reduced in CST-KO hearts, consistent with established roles in cardiomyopathy^79–86^. Ultrastructural analyses revealed myofibrillar disorganization, shortened sarcomeres, and disruption of A and I bands, changes that impair cross-bridge formation and contractility^87–90^. Concurrent mitochondrial abnormalities, including disrupted cristae architecture and increased autophagosomes, indicate compromised mitochondrial quality control. Given that ATP synthase is localized within cristae membranes, these structural defects likely further impair enzymatic efficiency in CST deficiency.

An additional dimension of CST action involves immune modulation. CST-KO hearts exhibit increased macrophage infiltration and altered cytokine profiles^24^. Transcriptomic analyses revealed dysregulation of inflammatory pathways, including reduced expression of anti-inflammatory genes (*Irak3*, *Ccl18*)^91,92^, and altered expression of innate immune receptors such as CD209 and TLR8. Changes in LILR family members^93^ and Vsig4^94^ further suggest context-dependent modulation of immune signaling. Because inflammation and mitochondrial dysfunction are closely coupled, CST’s combined immunomodulatory and metabolic effects likely act synergistically to preserve cardiac function.

Collectively, these findings position CST as an integrative regulator of cardiac metabolism, mitochondrial bioenergetics, sarcomeric organization, and immune homeostasis. Its absence leads to metabolic inflexibility, impaired ATP synthase activity, mitochondrial structural disruption, and contractile dysfunction. We propose a model in which CST enhances mitochondrial efficiency through interaction with the F₁-ATP synthase complex, linking endocrine peptide signaling to intrinsic regulation of cardiac energy metabolism.

Although biochemical, computational, and functional data support CST interaction with F₁-ATP synthase, high-resolution structural validation will further define the molecular interface. Future studies using cryo-electron microscopy, targeted mutagenesis of α–β catalytic residues, and quantitative biophysical approaches such as surface plasmon resonance or isothermal titration calorimetry will refine binding affinity, stoichiometry, and conformational effects. Importantly, the convergence of enzymatic, bioenergetic, and structural data strongly supports a functional interaction; additional studies are expected to refine mechanistic detail rather than alter the central conclusions.

CST emerges as a regulator of cardiac bioenergetics, structural integrity, and immune balance. By restoring metabolic flexibility, enhancing mitochondrial membrane potential, and increasing ATP synthase activity and ATP production, CST protects cardiomyocytes from lipotoxic and metabolic stress. These findings support a model in which CST modulates mitochondrial efficiency through interaction with the F₁-ATP synthase complex, highlighting its therapeutic potential in metabolic cardiomyopathy and HF.

## Methods

### Animals

Male WT and CST-KO (20-24 weeks old) on C57BL/6 J background were studied^24^. Animals were kept in a 12 hr dark/light cycle and fed a normal chow diet (NCD: 14% calorie from fat; LabDiet 5P00). All studies on animals were approved by the UCSD and Veterans Affairs San Diego Institutional Animal Care and Use Committees (IACUC) and conform to relevant National Institutes of Health guidelines.

### CST treatment

Mice were treated with saline or CST (1 µg/g body weight, intraperitoneal) for 4 weeks to evaluate its effect.

### RNA Sequencing

Total RNA was isolated from the left ventricle of C57BL6 WT (3), CST-KO (4), and CST-KO + CST mice (4) using the RNeasy kit (Qiagen, Germantown, MD). RNA was quantified using Nanodrop spectrophotometer (Thermo Fisher Scientific^TM^, Waltham, MA), and integrity was evaluated using Tapestation (Agilent Technologies, Santa Clara, CA). The following complementary DNA library preparation and sequencing were performed at the UCSD IGM core facility.

### Building a comprehensive database of heart disease datasets

Publicly available microarray and RNA-sequencing datasets were obtained from the National Center for Biotechnology Information (NCBI) Gene Expression Omnibus (GEO) repository^95–97^. For Affymetrix microarray platforms, raw intensity files were normalized using Robust Multichip Average (RMA)^98^. For RNA-sequencing datasets lacking normalized values, expression levels were quantified as transcripts per million (TPM)^99^ and log₂(TPM + 1) values were used for downstream analyses. In addition, we incorporated publicly available datasets normalized using alternative methods, including reads per kilobase per million mapped reads (RPKM)^100^, fragments per kilobase per million mapped reads (FPKM)^101^, TPM^102^, and counts per million (CPM)^103^. All single-cell RNAseq datasets were pseudo-bulked before the analysis. For Affymetrix microarray data, RMA normalization was preferred over MAS 5.0 due to its superior performance in reducing technical variability and improving cross-sample comparability^104^. Mouse heart RNA-seq data generated in this study (GSE301063) were processed using kallisto (version 0.45.0) and Ensembl GRCm38 annotation version 94 mouse genome. Publicly available datasets used in this study are cited with accession numbers in the manuscript, including GSE119087 and the Boolean Lab Heart Disease (BoLHD) dataset (Zenodo: 19722879)^105^.

### StepMiner analysis

StepMiner is a computational algorithm designed to identify step-wise transitions in ordered or time-series data by fitting step functions that minimize sum-of-squared errors^106^. The algorithm evaluates all possible step positions in a series and identifies the location corresponding to the sharpest transition between low and high expression states. For each candidate step position, StepMiner computes the mean expression levels on either side of the step and applies an adaptive regression scheme to select the step that best fits the data.

For a dataset containing *n* observations (*X*_1_ … *X*_*n*_), fitted values (*Ŷᵢ*) are estimated by assigning constant values before and after the step position *k*. The goodness of fit is assessed using an *F*-statistic defined as:

$$F=\frac{{\sum }_{i=1}^{n}{\left(\hat{{X}_{i}-\bar{X}}\right)}^{2}/(m-1)}{{\sum }_{i=1}^{n}{\left({X}_{i}-\hat{{X}_{i}}\right)}^{2}/(n-m)}$$

where *Xᵢ* represents observed values, *Ŷᵢ* represents fitted values, *m* denotes the degrees of freedom used in adaptive regression, and $\bar{X}=\frac{1}{n}{\sum }_{j=1}^{n}{X}_{j}$ is the mean expression level. The step position corresponding to the maximal F-statistic is selected as the optimal threshold, representing the gene expression switching point.

### Boolean analysis of gene expression data

Boolean analysis is a statistical framework that represents gene expression relationships using binary logic (high/low, 1/0) to infer invariant regulatory dependencies between gene pairs^26^. To perform Boolean analysis, continuous gene expression values were first discretized into binary states using the StepMiner algorithm. Expression values were sorted in ascending order, and a rising step function was fitted to determine a threshold. The midpoint of the step was used as the StepMiner threshold.

To reduce noise, a two-fold change margin (±0.5 around the StepMiner threshold) was applied, and samples falling within this intermediate zone were excluded from Boolean inference. After discretization, each gene pair (A, B) was classified into four Boolean quadrants: (low, low), (low, high), (high, low), and (high, high), with corresponding counts denoted as *a₀₀*, *a₀₁*, *a₁₀*, and *a₁₁*.

Boolean implication relationships were inferred by identifying sparsely populated quadrants using BooleanNet statistics^26,107^. The total number of samples was calculated as:

$${{\rm{total}}}={a}_{00}+{a}_{01}+{a}_{10}+{a}_{11}$$

The number of samples in which gene A or gene B was in the low state was computed as:

$${n}_{A,{{\rm{low}}}}={a}_{00}+{a}_{01},{n}_{B,{{\rm{low}}}}={a}_{00}+{a}_{10}$$

Expected counts ($\hat{n}$) for each quadrant were calculated assuming independence between gene A and gene B:

$$\hat{n}=\left(\frac{{n}_{A,{{\rm{low}}}}}{{{\rm{total}}}}\right)\left(\frac{{n}_{B,{{\rm{low}}}}}{{{\rm{total}}}}\right)\times {{\rm{total}}}$$

Quadrant sparsity was evaluated using a standardized statistic:

$${S}_{{ij}}=\frac{\hat{n}-n}{\sqrt{\hat{n}}}$$

and an associated error probability:

$${p}_{{ij}}=\frac{1}{2}\left(\frac{{a}_{{ij}}}{{a}_{{ij}}+{a}_{{ik}}}+\frac{{a}_{{ij}}}{{a}_{{ij}}+{a}_{{lj}}}\right)$$

A quadrant was considered sparse if *S*_*ij*_ > *sThr* and *p*_*ij*_ < *pThr*. Boolean implication relationships were defined based on the identity of the sparse quadrant(s). Symmetric relationships included Boolean equivalence (sparse off-diagonal quadrants) and Boolean opposition (sparse diagonal quadrants). Asymmetric relationships were inferred when a single quadrant was sparse, yielding implications such as *A low* → *B high*, *A high* → *B low*, and related forms.

Boolean implication relationships were identified using the criteria:

$${{\rm{Boolean}}}\,{{\rm{implication}}}=({S}_{{ij}} > {sThr},{p}_{{ij}} < {pThr})$$

For the large human dataset GSE119087, Boolean analysis was performed using thresholds *sThr* = *3* and *pThr* = *0.1*, consistent with previously validated BooleanNet parameters^104,106,108^. False discovery rates were estimated by random permutation of gene expression values within GSE119087, yielding *FDR* < *1e-4* under the selected thresholds^26^.

### Boolean network explorer (BoNE)

BoNE is an integrated computational framework for constructing, visualizing, and interrogating networks of progressive molecular changes underlying disease or biological processes. BoNE operates in three sequential steps.

First, gene expression values from each dataset were discretized into binary states (high or low) using the StepMiner algorithm. Second, pairwise gene–gene relationships were classified into one of six possible Boolean implication relationships (BIRs), comprising two symmetric relationships (equivalent and opposite) and four asymmetric relationships (low → low, low → high, high → high, high → low), which were represented as Boolean implication statements. Unlike conventional network inference approaches (e.g., correlation-based or Bayesian models) that rely on symmetric linear dependencies, BIRs explicitly encode directionality and are robust to sample heterogeneity arising from differences in genotype, phenotype, disease severity, ethnicity, and experimental perturbations. Because all samples conform to the same underlying logical constraints, Boolean implication relationships are highly reproducible across independent datasets.

Third, genes with similar expression architectures—defined by sharing at least 50% of equivalence relationships with other genes—were grouped into clusters and organized into a higher-order network. In the resulting Boolean implication network, clusters of genes represent nodes, and the dominant Boolean relationships between clusters define directed edges. BoNE enables unsupervised discovery of these structures while remaining agnostic to sample type or disease stage.

### Composite score for clusters of genes

To generate a composite score for each cluster, gene expression values within each cluster were first normalized and averaged. Normalization was performed using a modified *Z*-score centered on the StepMiner threshold, according to the formula:

$${\mathrm{Normalized}}\,{\mathrm{expression}}=\frac{\left({\mathrm{expr}} - S{\mathrm{Thr}}-0.5\right)}{3\times {\mathrm{SD}}}$$

where expr denotes the raw expression value, *S*Thr the StepMiner threshold, and SD the standard deviation.

A weighted linear combination of cluster averages was then used to compute a composite score for each sample. Sample-wise scores were visualized using color-coded bar plots and violin–swarm plots.

### Boolean analysis of RNA-seq data to assess CST regulation of gene expression in the heart

Boolean implication relationships were employed as a powerful tool to identify complex gene regulatory relationships influenced by CST. We employed a composite score (as described above)^27^, to construct a model of gene regulation in the absence of CST (CST-KO) and in the presence of CST (WT and CST-KO supplemented with CST). Given the observed increase in fibrosis and macrophage infiltration in CST-KO hearts, we focused on genes specifically expressed in fibroblasts, cardiomyocytes, and macrophages, utilizing Boolean analysis^26^.

For this analysis, we leveraged a large dataset comprising 25,955 human samples (GSE119087), profiled using Affymetrix Human U133 Plus 2.0 microarrays. To identify fibroblast- and cardiomyocyte-specific genes, we applied the Boolean Implication relationship DCN low → X low. To identify macrophage-specific genes, we applied the Boolean Implication relationship TYROBP low → X low. Boolean Implication relationships are discovered by using two parameters Sthr and pThr as described above^26^. Sthr = 3.0 and pthr = 0.1 are used to discover these Boolean Implication relationships, which have a false discovery rate < 1e-4.

Using these gene sets, we developed an algorithm to select a subset of genes for a composite score, ensuring adherence to the desired gene regulatory relationships—such as being downregulated in the absence of CST and upregulated in its presence or vice versa. The CST gene signatures were tested in other publicly available human (GSE141910, GSE5406, GSE302337, and GSE107480), mouse (GSE133054, GSE241771, GSE298801, GSE267693, and GSE234210), and rat (GSE144548 and GSE240921) datasets.

### In vivo tissue glucose uptake and metabolism

In vivo glucose uptake, G6P accumulation, and glycogen synthesis were assessed using dual radiotracer methodology with [³H]-glucose and [¹⁴C]-2-deoxyglucose (2DG) (NEN Life Science Products, PerkinElmer, Boston, MA, USA). Briefly, mice were fasted before tracer administration and then injected with a tracer mixture containing [³H]-glucose to assess glucose incorporation into glycogen and [¹⁴C]-2DG to quantify tissue glucose uptake. At the designated endpoint, mice were euthanized, and tissues were rapidly harvested, snap-frozen, and stored until analysis.

Left ventricular tissue was homogenized in ice-cold buffer, and aliquots were processed to determine total radiolabeled 2DG uptake, 2DG-6-phosphate accumulation, G6P-associated radioactivity, and glycogen incorporation. For assessment of glucose uptake, [¹⁴C]-2DG-derived radioactivity was measured in tissue lysates and normalized to tissue weight or protein content. For glycogen synthesis, tissue homogenates were subjected to alkaline digestion, glycogen precipitation, and repeated washing to remove unincorporated radiolabel. Radiolabeled glycogen was then quantified by scintillation counting.

Radioactivity from [³H] and [¹⁴C] channels was measured using a liquid scintillation counter with appropriate dual-label correction for isotope spillover. Glucose uptake and metabolic flux indices were calculated from tissue radioactivity after correction for background, tracer-specific activity, tissue mass, and plasma tracer availability, where applicable. Data are expressed relative to tissue weight or protein content and compared across experimental groups.

### GTT

Mice were fasted overnight for eight hours before starting the GTT. Blood glucose was measured from tail tip blood drops using a glucose strip (True Metrix, total diabetes, Whitestown, IN) and a glucometer (total diabetes, Whitestown, IN) after 15, 30, 60, 90, and 120 min of administration of intraperitoneal dextrose (2 g/kg). Data is presented as blood glucose concentration (mg/dL) over time and area under the curve above baseline^109^.

### PTT

All mice were fasted for eight hours before starting the PTT. Blood glucose was measured on a blood drop collected from the tail tip using a glucose strip and a glucometer at 0 min. Sodium pyruvate (2 g/kg intraperitoneally) was injected, followed by blood glucose measurements from the tail tip using a glucose strip and a glucometer after 15, 30, 60, 90, and 120 min after administration. Data is presented as blood glucose concentration (mg/dL) over time and area under the curve above baseline^109^.

### ITT

All mice were fasted for eight hours before starting the ITT. Blood glucose was measured on a blood drop collected from the tail tip using a glucose strip and a glucometer at 0 min. Human insulin (0.5 IU/kg intraperitoneally; Novolin, Novo Nordisk Inc., Plainsboro, New Jersey) was injected, followed by blood glucose measurements from the tail tip using a glucose strip and a glucometer after 15, 30, 60, 90, and 120 min after administration. Data is presented as blood glucose concentration (mg/dL) over time and area under the curve above baseline^109^.

### In vivo fatty acid incorporation, lipid extraction, and oxidation

In vivo fatty acid uptake, incorporation into lipids, and oxidation were assessed using radiolabeled palmitate. A 100 µl solution of [U-¹⁴C]-palmitic acid complexed to bovine serum albumin (BSA) (molar ratio 2.5:1), containing 1 µCi radioactivity and 250 µM palmitate, was administered intraperitoneally to each mouse. After 90 min, mice were euthanized, blood was collected, and tissues were rapidly harvested for analysis.

Left ventricular tissue (~100 mg) was homogenized in 0.8 ml of ice-cold 3.5 N perchloric acid. Lipids were extracted by adding 3 ml of methanol:chloroform (2:1, *v*/*v*), followed by vigorous vortexing^110^. Subsequently, 1.2 ml of 3.5 N perchloric acid was added to induce phase separation. After centrifugation, the lower organic (chloroform) phase containing radiolabeled lipids derived from [U-¹⁴C]-palmitate incorporation was collected. The upper aqueous acidic phase contained radiolabeled acid-soluble metabolites, reflecting fatty acid oxidation.

Radioactivity in both phases was quantified by liquid scintillation counting with appropriate correction for background. Lipid incorporation and oxidation were expressed relative to tissue weight or protein content.

### Protein analysis by immunoblotting

Left ventricular tissue was homogenized in ice-cold RIPA lysis buffer supplemented with protease and phosphatase inhibitor cocktails. Homogenates were clarified by centrifugation, and protein concentrations were determined using standard protein quantification assays. Equal amounts of protein were resolved by SDS–PAGE and transferred to PVDF membranes. Membranes were blocked and incubated with primary antibodies against phosphorylated AKT (Ser473-AKT Cat#9271S, Cell Signaling technology, Danvers, MA, USA); phosphorylated GSK-3β (Ser9-GSK-3β Cat#9832S, Cell Signaling technology); total AKT (AKT Cat# 4685S, Cell Signaling technology) and total GSK-3β (Cat#9832S, Cell Signaling Technology), as well as a Total OXPHOS Rodent WB Antibody Cocktail (Cat#110413, Abcam, Waltham, MA, USA). Following incubation with appropriate HRP-conjugated secondary antibodies, immunoreactive bands were detected using chemiluminescence. Band intensities were quantified by densitometry and normalized to appropriate loading controls or total protein levels, as indicated.

### Real-time PCR

Total RNA was isolated from left ventricular tissue using the RNeasy Mini Kit (Qiagen, Germantown, MD, USA) according to the manufacturer’s instructions. RNA was reverse-transcribed into cDNA using the qScript cDNA Synthesis Kit (Quantabio, Beverly, MA, USA). Quantitative real-time PCR (qPCR) was performed using PerfeCTa SYBR Green FastMix, Low ROX (Quantabio, Beverly, MA, USA) on an Applied Biosystems 7500 Fast Real-Time PCR System. Relative gene expression was calculated using the comparative Ct (ΔΔCt) method. Gene expression levels were normalized to the housekeeping gene *Rplp0*. Primer sequences are provided in Table 1.

**Table 1:** Primer sequences

Gene symbol	Gene ID	Forward Primer (5’ → 3’)	Reverse Primer (5’ → 3’)
*Actc1*	11464	CTGGATTCTGGCGATGGTGTA	CGGACAATTTCACGTTCAGCA
*Atp5pf*	11957	TATTGGCCCAGAGTATCAGCA	GGGGTTTGTCGATGACTTCAAAT
*Cd36*	12491	ATGGGCTGTGATCGGAACTG	GTCTTCCCAATAAGCATGTCTCC
*Cers2*	76893	CCTTCTACTGGTCCCTGCTCTT	TGGCAAACCAGGAGAAGCAGAG
*Dgat2*	67800	GCGCTACTTCCGAGACTACTT	GGGCCTTATGCCAGGAAACT
*Map1lc3b*	67443	TTATAGAGCGATACAAGGGGGAG	CGCCGTCTGATTATCTTGATGAG
*Myl4*	17896	AAGAAACCCGAGCCTAAGAAGG	TGGGTCAAAGGCAGAGTCCT
*Myl7*	17898	GGCACAACGTGGCTCTTCTAA	TGCAGATGATCCCATCCCTGT
*Ndufa3*	66091	ATGGCCGGGAGAATCTCTG	AGGGGCTAATCATGGGCATAAT
*Pdk2*	18604	AGGGGCACCCAAGTACATC	TGCCGGAGGAAAGTGAATGAC
*Pfkm*	18642	TGTGGTCCGAGTTGGTATCTT	GCACTTCCAATCACTGTGCC
*Pink1*	68943	TTCTTCCGCCAGTCGGTAG	CTGCTTCTCCTCGATCAGCC
*Ppara*	19013	AGAGCCCCATCTGTCCTCTC	ACTGGTAGTCTGCAAAACCAAA
*Prkn*	50873	TCTTCCAGTGTAACCACCGTC	GGCAGGGAGTAGCCAAGTT
*Rplp0*	11837	AGATTCGGGATATGCTGTTGGC	TCGGGTCCTAGACCAGTGTTC
*Sdhc*	66052	GCTGCGTTCTTGCTGAGACA	ATCTCCTCCTTAGCTGTGGTT
*Slc27a1*	26457	CGCTTTCTGCGTATCGTCTG	GATGCACGGGATCGTGTCT
*Slc27a2*	26458	TCCTCCAAGATGTGCGGTACT	TAGGTGAGCGTCTCGTCTCG
*Slc2a4*	20528	GTGACTGGAACACTGGTCCTA	CCAGCCACGTTGCATTGTAG
*Sptlc1*	268656	ACGAGGCTCCAGCATACCAT	TCAGAACGCTCCTGCAACTTG
*Tnni3*	21954	TCTGCCAACTACCGAGCCTAT	CTCTTCTGCCTCTCGTTCCAT
*Tnnt2*	21956	CAGAGGAGGCCAACGTAGAAG	CTCCATCGGGGATCTTGGGT
*Tpm*	22003	CAGAAGGCAAATGTGCCGAG	TCCAGCATCTGGTGCATACTA
*Ttn*	22138	GACACCACAAGGTGCAAAGTC	CCCACTGTTCTTGACCGTATCT

### Transmission electron microscopy (TEM)

To remove blood and prepare tissues for fixation, mice were cannulated through the apex of the heart and perfused with a Ca²⁺- and Mg²⁺-free buffer consisting of Dulbecco’s phosphate-buffered saline (DPBS) supplemented with 9.46 mM KCl to arrest the heart in diastole^111^. Following perfusion, left ventricular tissue was fixed, processed, embedded, sectioned, and stained using standard TEM procedures. Ultrathin sections were mounted on grids and imaged using a JEOL JEM-1400 Plus transmission electron microscope (JEOL, Peabody, MA, USA). Images were acquired with a Gatan OneView digital camera at 4k × 4k resolution (Gatan, Pleasanton, CA, USA).

### Morphometric analysis

Sarcomere length was measured from electron micrographs using ImageJ software. Mitochondrial cristae membrane surface area was quantified by manually tracing individual cristae within each mitochondrion. The total cristae membrane surface area was normalized to the outer mitochondrial membrane surface area for each mitochondrion to account for differences in mitochondrial size^111^.

### Ligand affinity isolation of the CST binding protein

Mouse heart tissue was homogenized in ice-cold buffer (10 mM HEPES, pH 7.4, 0.1 mM EDTA) supplemented with a protease inhibitor cocktail (Protease Inhibitor Cocktail Set III, Calbiochem, San Diego, CA, USA; used at 1× final concentration, including PMSF 5 mM, benzamidine hydrochloride 50 µg/ml, and TLCK 0.1 mM). Homogenates were centrifuged at 18,000 rpm for 20 min at 4 °C to pellet crude membranes. The membrane pellet was washed once with the same buffer and then solubilized for 2 h at 4 °C in solubilization buffer (25 mM HEPES, pH 7.4, 100 mM NaCl, 2 mM MgCl₂, 1 mM KCl, 1% (*v*/*v*) Triton X-100, and protease inhibitors). The mixture was centrifuged at 18,000 rpm for 1 h at 4 °C, and the supernatant was collected as the solubilized membrane fraction.

Biotinylated human catestatin (CST) peptide was synthesized with a C-terminal biotin tag separated by a four–amino acid spacer (SGSG). A ligand affinity matrix was prepared by incubating 5 mg of biotinylated CST with 1 ml of 50% streptavidin–agarose resin (Thermo Fisher Scientific, Waltham, MA, USA) in column buffer (25 mM HEPES, pH 7.4, 100 mM NaCl, 2 mM MgCl₂, 1 mM KCl, 1% Triton X-100, and protease inhibitors) for 1 h at 4 °C with gentle mixing.

The resin was washed extensively and incubated overnight at 4 °C with the solubilized membrane fraction. After binding, the resin was packed into a chromatography column and washed with column buffer containing 0.1% Triton X-100 to remove non-specifically bound proteins. Bound proteins were eluted using column buffer adjusted to pH 5.5. Eluted fractions were precipitated with trichloroacetic acid (TCA), resolved by 10% SDS–PAGE, and visualized by Coomassie Brilliant Blue G-250 staining (SimplyBlue SafeStain, Invitrogen, Waltham, MA, USA). A prominent protein band (~53 kDa) was excised for downstream analysis.

### In-gel digest of the CST-binding protein and LC-tandem-MS/MS analysis

Excised gel bands were subjected to in-gel digestion with trypsin. The resulting peptides were separated by reverse-phase liquid chromatography on a C18 column and analyzed by tandem mass spectrometry using electrospray ionization on a QSTAR Elite hybrid mass spectrometer (AB Sciex, Framingham, MA, USA) coupled to a nanoscale reversed-phase high-performance liquid chromatography system (Tempo). Peptides were resolved on a 10 cm × 180 µm inner diameter capillary column packed with 5 µm C18 Zorbax beads (Agilent Technologies, Santa Clara, CA, USA). Data acquisition and analysis were performed using standard manufacturer-recommended settings.

### CST modeling

CST is a 21–amino acid endogenous cationic peptide with predominantly hydrophobic characteristics. The initial structure of human CST was obtained from the Protein Data Bank (PDB ID: 1LV4)^112^. To explore its conformational properties, the structure was first subjected to energy minimization to optimize side-chain positioning. The minimized structure was then used as the starting point for MD simulations in an explicit solvent environment. MD simulations were performed for 250 ns to generate a conformational ensemble of CST^113^. Representative structures from the equilibrated trajectory were used for downstream structural analyses and protein–protein docking studies.

### F1-ATP synthase modeling

ATP synthase is a multi-subunit enzyme responsible for ATP production and consists of two functional domains, F₀ and F₁. Based on experimental evidence indicating interaction of CST with the α/β interface of the F₁ domain, a structural model of mouse F₁-ATP synthase was generated. A homology model was constructed using the crystal structure of bovine F₁-ATP synthase (PDB ID: 1E79) as the template^53^, given the high sequence similarity between the two species. Initial model building was performed using MODELLER (version 9.13)^114^. The model included bound nucleotides consistent with the template structure, comprising two ATP and three ADP molecules. The modeled structure was subjected to sequential energy minimization and equilibration steps, followed by a 100 ns MD simulation in explicit solvent to obtain a stable and refined conformation. The final equilibrated structure was used for subsequent protein–protein docking analyses with CST.

### Protein-protein docking of CST on F1-ATP synthase

To investigate the molecular interaction between CST and F₁-ATP synthase, protein–protein docking was performed using the HEX docking algorithm. Docking was conducted in an unbiased manner, allowing CST to sample the entire surface of the F₁-ATP synthase structure to identify potential binding sites. Docked complexes were ranked based on the HEX scoring function, and the top-scoring complex was selected for further analysis. The selected CST–F₁-ATP synthase complex was subsequently subjected to MD simulation in an explicit solvent environment for 100 ns to refine the interaction and assess structural stability.

### MD Simulation methodology

MD simulations were performed using the GROMACS (version 4.5.5)^115^ simulation package with the CHARMM36 force field^116,117^. Initial structures were subjected to energy minimization using steepest descent followed by conjugate gradient algorithms (1000 steps each) to remove steric clashes and optimize geometry. The minimized system was solvated in a cubic periodic simulation box containing approximately 180,000 explicit water molecules modeled using the TIP3P water model. Sodium and chloride ions were added to achieve a physiological ionic strength of 0.15 M and to neutralize the system. The solvated system was further energy minimized prior to equilibration.

The system was gradually heated to 310 K and equilibrated under constant pressure and temperature (NPT ensemble) at 1 atm and 310 K for 10 ns, allowing stabilization of temperature, pressure, and solvent density. This was followed by a 100 ns production simulation, from which all analyses were derived. Long-range electrostatic interactions were treated using the Particle Mesh Ewald (PME) method with a real-space cutoff of 1.0 nm. All bonds involving hydrogen atoms were constrained using the SHAKE algorithm. Structural visualization and rendering were performed using visual molecular dynamics (VMD)^118^, and protein–protein interaction interfaces were analyzed using PDBsum^119^.

### AlphaFold modeling of CST-bound F1-ATP synthase complex

Amino acid sequences for mouse F₁-ATP synthase subunits α (UniProt: Q03265), β (UniProt: P56480), and γ (UniProt: Q91VR2), along with the CST peptide, were retrieved from UniProt. Sequences were formatted for multimeric structure prediction using AlphaFold in multimer mode to model interactions between CST and the α, β, and γ subunits of F₁-ATP synthase. To approximate the functional state of the complex, nucleotide ligands (ATP and ADP) were included in the model consistent with the known catalytic configuration of the F₁ domain. Predicted structural models were evaluated and visualized using UCSF ChimeraX^120^.

### Cellular uptake and localization of CST

Primary cardiomyocytes were isolated from 2-day-old C57BL/6 mice using the Pierce Primary Cardiomyocyte Isolation Kit (Thermo Fisher Scientific, Waltham, MA, USA) according to the manufacturer’s instructions. Cells were cultured until spontaneous contractions were observed (approximately 7 days post-isolation). Cardiomyocytes were incubated with 5 µM fluorescein isothiocyanate (FITC)-labeled CST (Bon Opus Biosciences, Millburn, NJ, USA) for 4 h. To visualize mitochondria, MitoTracker Deep Red FM (500 nM; Thermo Fisher Scientific) was added 30 min prior to fixation. Cells were then fixed with 4% paraformaldehyde and counterstained with DAPI to label nuclei. Fluorescence imaging was performed using a Keyence fluorescence microscope at 40× magnification.

### Colocalization of CST and mitochondria

H9c2 cells were plated on chamber slides and cultured overnight. Cells were incubated with MitoTracker Deep Red FM (500 nM; Thermo Fisher Scientific, Waltham, MA, USA) for 30 min at 37 °C to label mitochondria. Following incubation, cells were washed with phosphate-buffered saline (PBS) and fixed with 4% paraformaldehyde for 15 min at room temperature. Fixed cells were permeabilized with PBS containing 0.1% Triton X-100 for 10 min and then incubated overnight at 4 °C with primary antibody against CST diluted in permeabilization buffer. The following day, cells were washed with PBS and incubated with Alexa Fluor 488–conjugated secondary antibody for 1 h at room temperature in the dark. Nuclei were stained with Hoechst dye for 10 min. After washing, slides were mounted with antifade mounting medium.

Fluorescence images were acquired using a Keyence fluorescence microscope at 40× magnification with identical acquisition settings across conditions. Colocalization analysis was performed using Fiji (ImageJ). Regions of interest were segmented using Cellpose, and colocalization metrics were calculated using the JACoP plugin. Pearson’s correlation coefficient and Manders’ overlap coefficients were determined using automatic thresholding based on Costes’ method.

### Determination of mitochondrial membrane potential

Primary cardiomyocytes were isolated from 2-day-old C57BL/6 mice using the Pierce Primary Cardiomyocyte Isolation Kit (Thermo Fisher Scientific, Waltham, MA, USA) according to the manufacturer’s instructions. Cells were cultured until spontaneous contractions were observed (approximately 7 days post-isolation). Cardiomyocytes were treated with catestatin (CST; 1 µM) or saline control for 4 h. Mitochondrial membrane potential was assessed using Image-iT TMRM reagent (Invitrogen, Waltham, MA, USA) at a final concentration of 100 nM, added during the last 30 min of treatment. Cells were then washed with PBS, lysed with 150 µl of cell lysis buffer per well, and fluorescence intensity was measured using a plate reader (excitation/emission: 548/574 nm).

### ATP synthase (complex V) activity assay

Control and catestatin (CST; 1 µM)-treated H9c2 rat cardiomyocytes were cultured for 24 h prior to mitochondrial isolation. Cells were washed with PBS, harvested, and pelleted by centrifugation. The cell pellet was resuspended in ice-cold isolation buffer 1 (30 mM Tris-HCl, pH 7.4, 225 mM mannitol, 75 mM sucrose, 0.1 mM EGTA) and homogenized using a Dounce homogenizer on ice. The homogenate was centrifuged at 600×*g* for 5 min at 4 °C to remove nuclei and unbroken cells. The supernatant was further centrifuged at 7000×*g* for 10 min at 4 °C to pellet mitochondria. The mitochondrial pellet was washed twice with isolation buffer 2 (225 mM mannitol, 75 mM sucrose, 30 mM Tris-HCl, pH 7.4) by centrifugation at 10,000×*g* for 10 min at 4 °C. The final pellet was resuspended in mitochondrial resuspension buffer (250 mM mannitol, 0.5 mM EGTA, 5 mM HEPES, pH 7.4) and stored at −80 °C until analysis.

ATP synthase (Complex V) activity was measured using a microplate-based assay kit (Abcam, ab109716) according to the manufacturer’s instructions. Enzymatic activity was determined spectrophotometrically by monitoring the decrease in absorbance at 340 nm, corresponding to NADH oxidation^121^.

Complex V activity was calculated using the following equation:

$${{\rm{Complex}}}\,{{\rm{V}}}\,{{\rm{activity}}}=\frac{\Delta {A}_{340}/\min \times 1000}{\varepsilon \times l\times V\times [{{\rm{protein}}}]}$$

where Δ*A*_340_/min is the rate of absorbance change, ε=6.22 ${{\mbox{mM}}}^{-1}\cdot {{\mbox{cm}}}^{-1}$ is the extinction coefficient of NADH, *l* is the path length (cm), *V* is the sample volume (ml), and protein concentration is expressed in mg/ml.

### Determination of ATP generation

Primary cardiomyocytes were isolated from 2-day-old C57BL/6 mice using the Pierce Primary Cardiomyocyte Isolation Kit (Thermo Fisher Scientific, Waltham, MA, USA) according to the manufacturer’s instructions. Cells were cultured until spontaneous contractions were observed (approximately 7 days post-isolation). Cardiomyocytes were treated with catestatin (CST; 1 µM) or saline control for 24 h. Intracellular ATP levels were quantified using a firefly luciferase-based ATP assay kit (#28854, Cell Signaling Technology, Danvers, MA, USA) according to the manufacturer’s protocol. Luminescence was measured using a microplate reader and used as an index of ATP content.

### Mitochondrial metabolism (Seahorse assay)

H9c2 cardiomyocytes were seeded at a density of approximately 15,000 cells per well in Seahorse XF assay plates and incubated overnight in DMEM. The following day, cells were incubated in Seahorse assay medium and treated with catestatin (CST; 0.5–10 µM) for 1 h at room temperature. Mitochondrial function was assessed using a Seahorse XF Analyzer by measuring the oxygen consumption rate (OCR). A mitochondrial stress test was performed with sequential injections of oligomycin (1.5 µM), FCCP (1.5 µM), and rotenone/antimycin A (0.5 µM) to evaluate basal respiration, ATP-linked respiration, maximal respiratory capacity, and non-mitochondrial respiration.

Following the assay, OCR values were normalized to the cell number determined by Hoechst staining using a Cytation 5 imaging system. OCR data are presented as pmol O₂/min/1000 cells. Mitochondrial parameters were calculated from OCR traces using standard analytical methods. An ATP rate assay was performed in parallel using the same seeding density (15,000 cells per well) according to the manufacturer’s protocol. ATP production rates were normalized to cell number and expressed as OCR-derived values (pmol/min/1000 cells)^122^.

### Illustrations

All illustrations were created by the authors using PRISM/PowerPoint/NIH BioArt; no third-party copyrighted materials were used.

### Statistics and reproducibility

All data are presented as mean ± SEM, and individual data points are shown. Sample size (*n*) for each experimental group is specified in the corresponding figure or figure legend. For Figs. 3F and 6Q–T, morphometric analyses of glycogen granules and mitochondria were performed on 15 sections per animal from 3 biologically independent animals. Statistical analyses were performed using GraphPad Prism (version 10.4.0; Boston, MA, USA). Data were analyzed using repeated-measures (RM) two-way ANOVA or two-way ANOVA, followed by appropriate post hoc tests and pairwise comparisons. For comparisons between two groups, a two-tailed unpaired Student’s *t*-test or Mann–Whitney *U*-test was used, depending on normality assessed by the Shapiro–Wilk test. Where appropriate, equality of variances was assessed (e.g., Levene’s test), and statistical approaches were adjusted accordingly. A significance threshold of α < 0.05 was used. All measurements were obtained from biologically independent samples unless otherwise specified in the figure or figure legends.

### Reporting summary

Further information on research design is available in the Nature Portfolio Reporting Summary linked to this article.

## Supplementary information

Supplementary Information

Description of Additional Supplementary Files

Supplementary Data 1

Supplementary Data 2

Reporting Summary

Transparent Peer Review file

## Data Availability

The RNA-seq data generated in this study are publicly available in the GEO under accession number GSE301063. All other datasets analyzed are available from publicly accessible repositories as cited in ref. ^105^. Source data are provided as Supplementary Data 2. Additional data are available from the corresponding author upon reasonable request.

## Code availability

Code used for reproducing analyses and generating Figs. 1 and 2 is publicly available at GitHub (https://github.com/sahoo00/Heart; CST-KO_Heart.ipynb)^123^. A version of the code has been archived in a DOI-minting repository (e.g., Zenodo) and the DOI is cited in the main reference list to ensure long-term accessibility and reproducibility. The code includes all necessary scripts and parameters required to reproduce the reported results.
