# Supplementary material for: Catestatin restores cardiac metabolic flexibility and enhances mitochondrial function
Source: Commun Biol. 2026 May 23;9:1110. doi: 10.1038/s42003-026-10310-z (PMC13483899; doi:10.1038/s42003-026-10310-z)
Supplement: Supplementary file 1 [file 42003_2026_10310_MOESM1_ESM.pdf]

Catestatin restores cardiac metabolic flexibility and enhances mitochondrial function

Satadeepa Kal[1,2*], Venkat R. Chirasani[3*#], Nilima Biswas[2], Sumana Mahata[4], Suborno Jati[2], Kechun Tang[1], Teresa Pasqua[5,] Ennio Avolio[6], Ramamurthy Chitteti[2,8], Shrabastee Chakraborty[7], Gautam Bandyopadhyay[2], Brian P. Head[2,8], Geert van den Bogaart[9], Hemal H. Patel[2,8†], Debashis Sahoo[2†], Sanjib Senapati[3†], and Sushil K. Mahata[2,8†]

[1]Veterans Medical Research Foundation, San Diego, CA, USA; [2]University of California San Diego, La Jolla, CA, USA; [3]Department of Biotechnology, Indian Institute of Technology Madras, Chennai, India; [4]Riverside University Health System, Riverside, CA, USA; [5]University Magna Graecia of Catanzaro, Catanzaro, Italy; [6]University of Calabria, Cosenza, Italy; [7]University of Michigan, Ann Arbor, MI, USA; [8]VA San Diego Healthcare System, San Diego, CA, USA; [9]University of Groningen, Groningen, The Netherlands. [#]Present address: Department of Biochemistry and Biophysics, R. L. Juliano Structural Bioinformatics Core, University of North Carolina at Chapel Hill, Chapel Hill, NC, USA.

* Equal contribution

† Senior corresponding authors

Short title: Catestatin regulation of metabolic flexibility and mitochondrial function

Key words: Catestatin, diabetic heart, substrate metabolism, ATP synthase, mitochondrial membrane potential, ATP synthesis

Address correspondences to:

Sushil K. Mahata, Ph.D.
Metabolic Physiology & Ultrastructural Biology Laboratory
Department of Medicine
University of California, San Diego (0732)
9575 Gilman Drive
La Jolla, CA 92093-0732
Tel (858)-552-8585, extension 2637
E-mail: smahata@health.ucsd.edu
ORCID ID: 0000-0002-9154-0787

or Sanjib Senapati (sanjibs@iitm.ac.in; ORCID ID: 0000-0002-6671-8299), or Debashis Sahoo (dsahoo@health.ucsd.edu; ORCID ID:0000-0003-2329-8228), or Hemal Patel (hepatel@health.ucsd.edu; ORCID ID: 0000-0001-6722-9625)

S-Fig. 1. CST deficiency increases cardiac mitophagy via the PINK1–Parkin pathway, which is reversed by CST supplementation

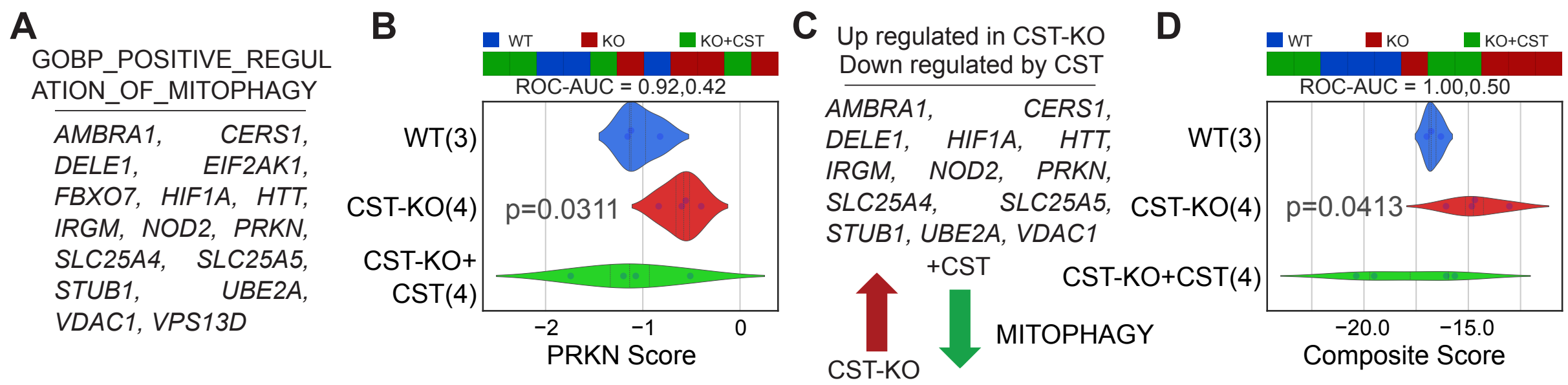

(A) The MSigDB gene set GOBP_POSITIVE_REGULATION_OF_MITOPHAGY, consisting of 16 genes (*AMBRA1*, *CERS1*, *DELE1*, *EIF2AK1*, *FBXO7*, *HIF1A*, *HTT*, *IRGM*, *NOD2*, *PRKN*, *SLC25A4*, *SLC25A5*, *STUB1*, *UBE2A*, *VDAC1*, *VPS13D*) was used for this analysis. **(B)** Bar and violin plots illustrating expression of the individual gene *PRKN* (aka *PARK2*), which is upregulated in CST-KO hearts; CST treatment reverses this effect. **(C)** A subset of 13 genes from the GOBP_POSITIVE_REGULATION_OF_MITOPHAGY signature was selected for downstream scoring. **(D)** Bar and violin plots of the composite score generated from the 13 selected genes (panel C), demonstrating increased mitophagy signature in CST-KO hearts, which is reversed by CST treatment.

S-Fig. 2. CST deficiency disrupts sarcomere gene expression and mitochondrial gene programs, both of which are rescued by CST supplementation

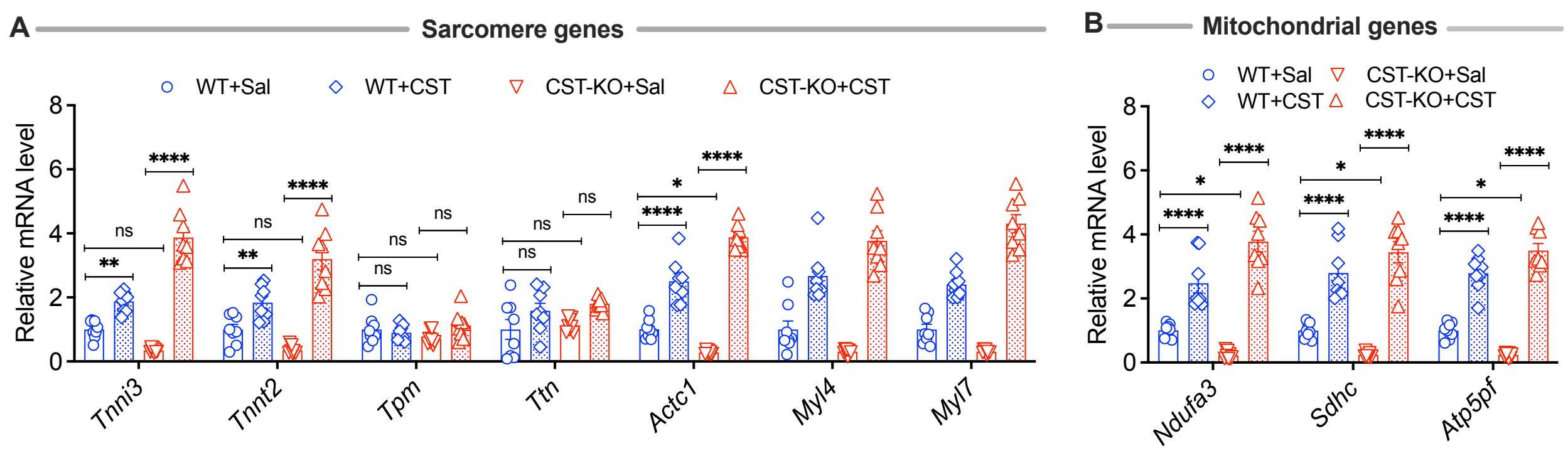

(A) qRT-PCR analysis of key sarcomeric structural genes (*Tnni3, Tnnt2, Tpm, Ttn, Actc1, Myl4, Myl7*) in left ventricular tissue from WT+Sal, WT+CST, CST-KO+Sal, and CST-KO+CST mice. CST-KO hearts showed marked downregulation of several sarcomere genes compared with WT, consistent with impaired contractile architecture. CST supplementation significantly restored the expression of *Tnni3, Tnnt2, Tpm,* and *Ttn*, indicating recovery of sarcomeric integrity. Genes with no significant change are indicated (ns). **(B)** Mitochondrial gene expression profiling revealed significant reductions in oxidative phosphorylation (OXPHOS) components in CST-KO hearts, including *Ndufa3* (Complex I), *Sdhc* (Complex II), and *Atp5pf* (Complex V). CST treatment robustly increased the expression of all three mitochondrial genes toward WT levels, demonstrating a rescue of mitochondrial biogenesis and respiratory chain transcriptional programs. Significance is indicated as: *$p < 0.05$, **$p < 0.01$, ****$p < 0.0001$.

S-Fig. 3. Identification of a CST binding protein from mouse heart employing affinity chromatography followed by LC-tandem-MS/MS mass spectrometry

A Protein sequence of ATP synthase beta subunit

MLSLVGRVASASASGALRGLSPSAALPQAQLLLRAAPAGVHPARDYAAQASAAPKAGTATGRIVAVIGAVVDVQFDEGLPPILNALEVQGRDS
RLVLEVAQHLGESTVRTIAMDGTEGLVRGQKVLDSGAPIKIPVGPETLGRIMNVIGEPIDERGPIKTKQFAPIHAEAPEFIEMSVEQEILVTG
IKVVDLLAPYAKGGKIGLFGGAGVGKTVLIMELINNVAKAHGGYSVFAGVGERTREGNDLYHEMIESGVINLKDATSKVALVYGQMNEPPGAR
ARVALTGLTVAEYFRDQEGQDVLLFIDNIFRFTQAGSEVSALLGRIPSAVGYQPTLATDMGTMQERITTTKKGSITSVQAIYVPADDLTDPAP
ATTFAHLDATTVLSRAIAELGIYPAVDPLDSTSRIMDPNIVGNEHYDVARGVQKILQDYKSLQDIIAILGMDELSEEDKLTVSRARKIQRFLS
QPFQVAEVFTGHMGKLVPLKETIKGFQQILAGEYDHLPEQAFYMVGPIEEAVAKADKLAEEHGS

B One representative MS/MS spectrum to identify ATP synthase beta subunit from amino acids 226 to 239

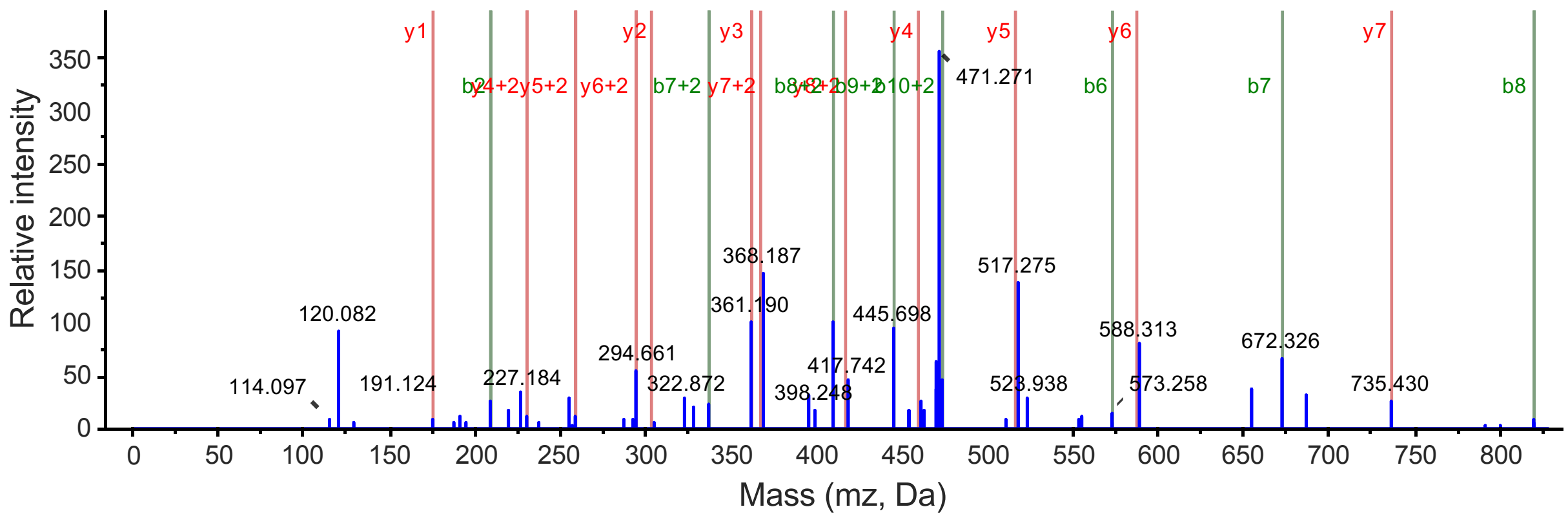

(**A**) Protein sequence coverage map of mouse ATP synthase subunit b. (**B**) Mouse ATP synthase α-subunit protein sequence coverage map by fragments of a CST-binding protein identified by trypsin-LC-MS/MS; colors are based on peptide confidence: green, peptide has been identified with at least 95% confidence; purple, peptide identified with at least 50% confidence. Regions identified with lower confidence are shown in red. MS/MS spectrum of precursor mass 1286.7 generated after trypsin digestion of CST bound protein.

S-Fig. 4. Residue–level interactions between CST and F1-ATPase

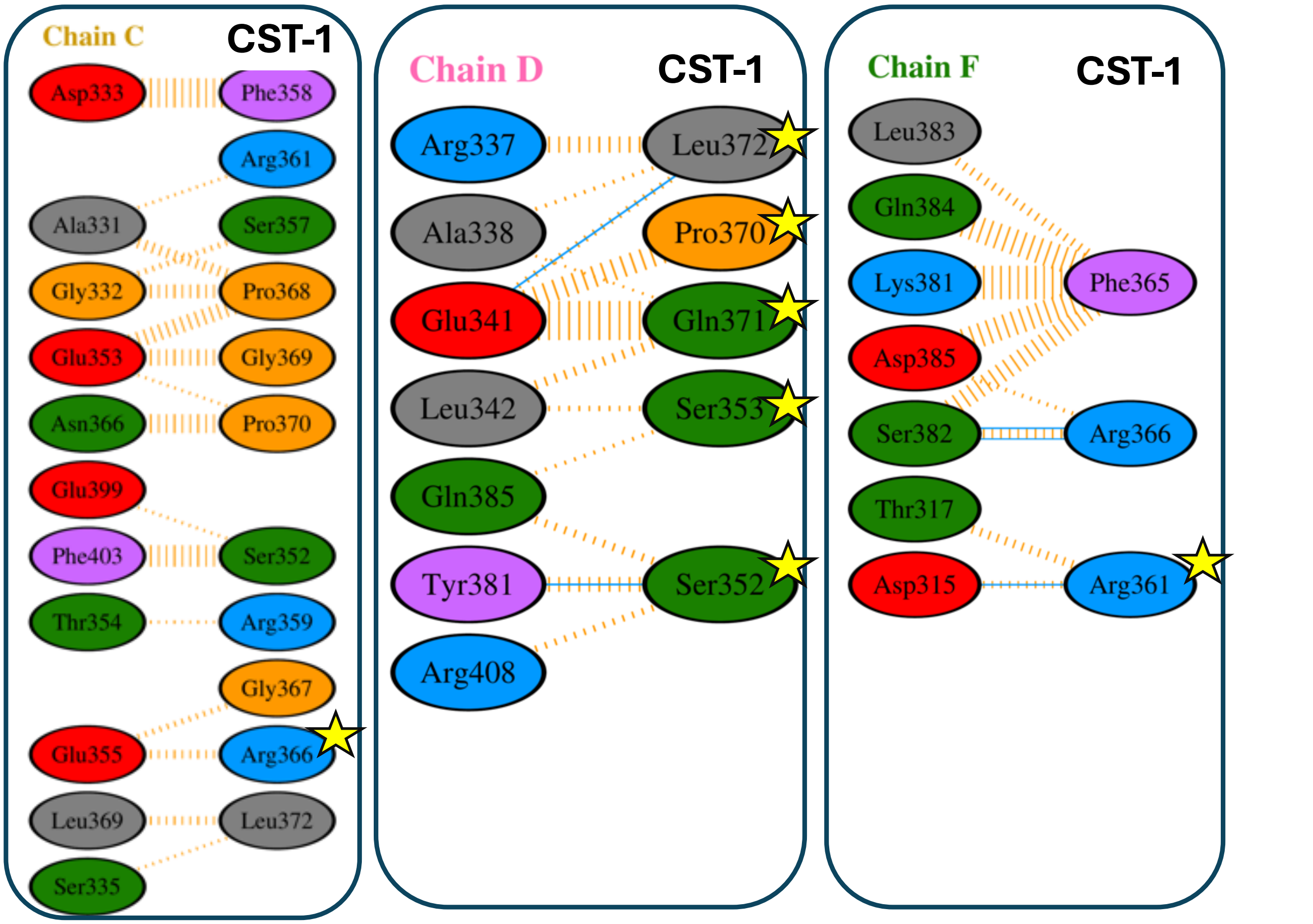

Interactions of CST with F1-ATPase is depicted. Interactions of three other CST molecules which also can bind to the α-β interface of F1-ATPase, as obtained from progressive protein-protein docking study, are shown subsequently in S-Fig 5. Interestingly, a set of seven CST residues was found to be common in all four CST-F1-ATPase complexes. These residues are highlighted by yellow stars in S-Fig 4 and S-Fig 5. Potential hydrogen bonds between ATPase and CST residues are represented by blue lines and hydrophobic contacts are represented by orange broken lines. The color code given to each residue is based on its nature: positively charged residues in cyan; negatively charged residues in red; neutral charged residues in green; aliphatic residues in grey; aromatic residues in pink; PRO and GLY in orange; CYS in yellow..

S-Fig. 5. Residue -level interactions between CST and F1-ATPase

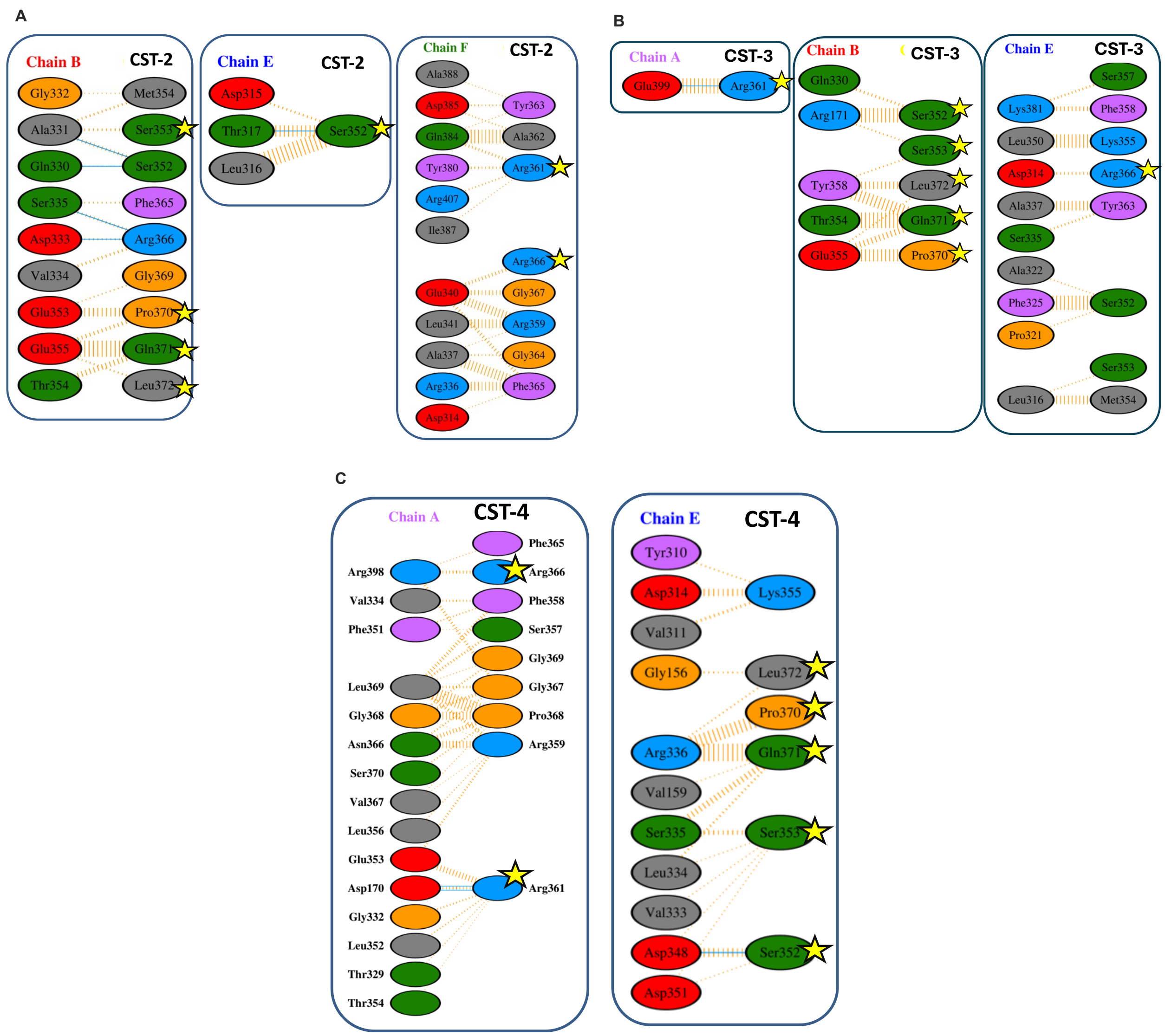

Interactions of (**A**) CST-2, (**B**) CST-3, and (**C**) CST-4 with F1-ATPase are depic color scheme is similar to S-Fig 4.

S-Fig. 6. Cell permeable CST co-localizes with mitochondria in cardiomyocytes

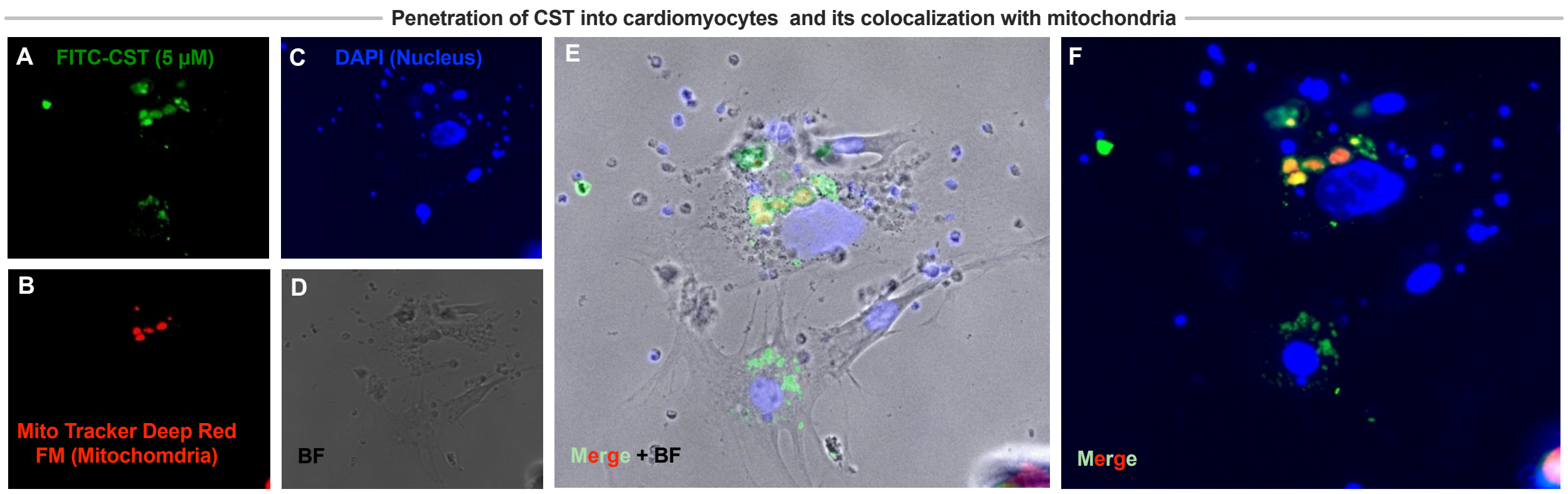

Confocal micrographs of: (**A**) Cardiomyocytes incubated with FITC-CST, (**B**) Mito Tracker Deep Red FM, and (**C**) DAPI. (**D**) Bright field (BF) photograph. (**E**) Merged plus bright field. (**F**) merged.

S-Fig. 7. Data summary of Seahorse XF Cell Mito Stress Test assays

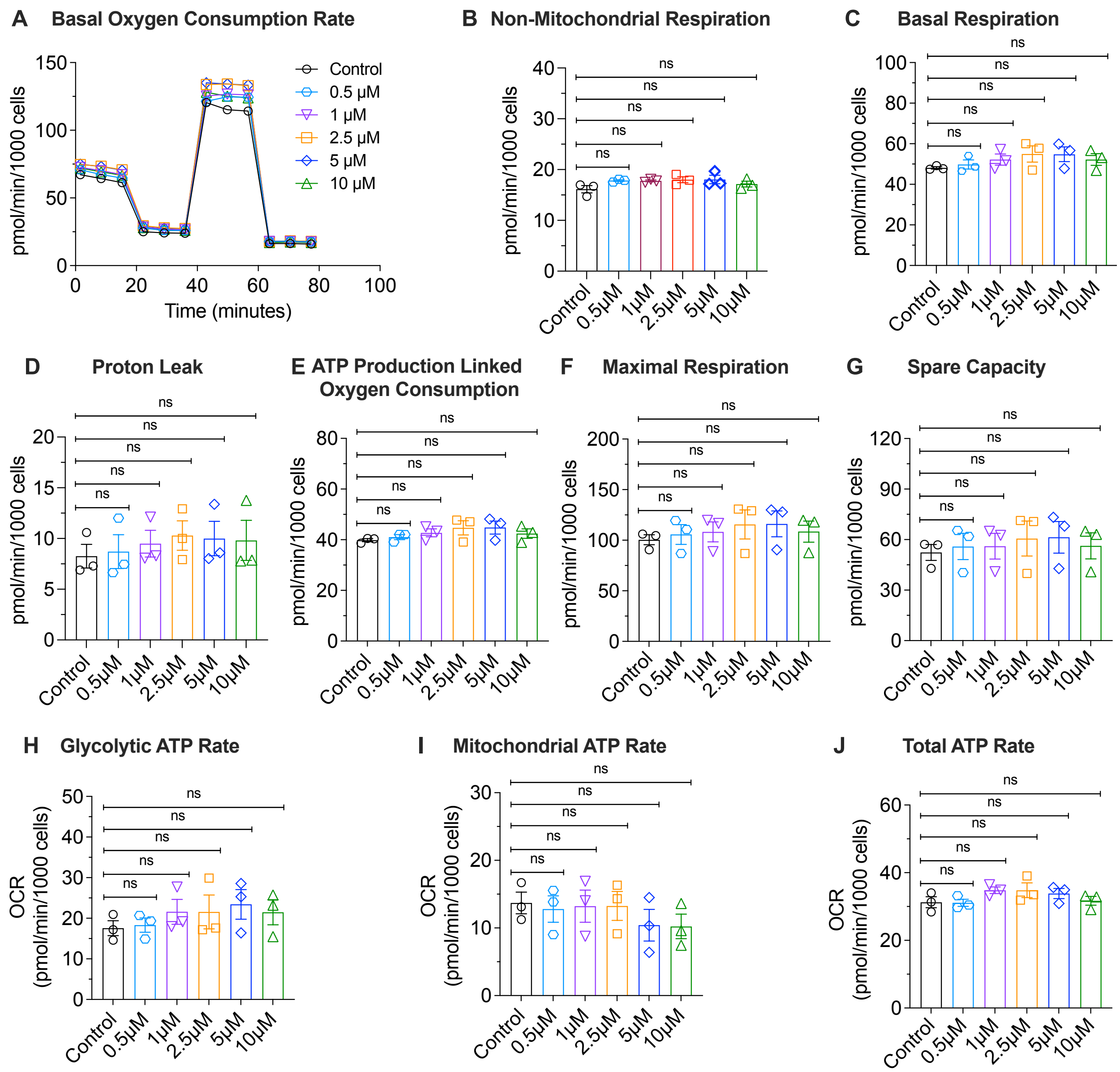

(**A**) Profiles of Mito Stress Test data for OCR, n = 3 biological replicates with multiple technical replicates H9c2 cell lines with injections into media of the specific stressors oligomycin (Olig.), carbonyl cyanite-4 (trifluoromethoxy) phenylhydrazone (FCCP), and Rotenone/Antimycin A (R/A). (**B**) Basal respiration. (**C**) Non-Mitochondrial respiration. (**D**) Proton leak. (**E**) ATP production linked OCR. (**F**) Maximal Respiration. (**G**) Spare capacity. Experiments were performed in n = 3 replicates. *p*<0.05; One-way ANOVA compared with control.
