# Supplementary material for: Catestatin restores cardiac metabolic flexibility and enhances mitochondrial function
Source: Commun Biol. 2026 May 23;9:1110. doi: 10.1038/s42003-026-10310-z (PMC13483899; doi:10.1038/s42003-026-10310-z)
Supplement: Supplementary file 2 [file 42003_2026_10310_MOESM2_ESM.pdf]

Description of Additional Supplementary Files

File name: Supplementary Data 1
Description: Summary of the peptides from Mass Spec data

File name: Supplementary Data 2
Description: Numerical and other Source data for the Main Figure 3, 4, 5, 6, and 8.
