# Supplementary material for: Catestatin restores cardiac metabolic flexibility and enhances mitochondrial function
Source: Commun Biol. 2026 May 23;9:1110. doi: 10.1038/s42003-026-10310-z (PMC13483899; doi:10.1038/s42003-026-10310-z)
Supplement: Supplementary file 4 [file 42003_2026_10310_MOESM6_ESM.pdf]

nature portfolio

Peer Review File

Catestatin improves cardiac metabolic flexibility by reversing insulin resistance and potentiates mitochondrial function

Corresponding Author: Professor Sushil Mahata

This file contains all reviewer reports in order by version, followed by all author rebuttals in order by version.

Version 0:

Reviewer comments:

Reviewer #1

(Remarks to the Author)
In this manuscript, Chirasani et al. evaluate the role of Catestatin (CST) in cardiac metabolic flexibility and mitochondrial function. In the studies, the Authors identify CST as a binding partner to F1 ATP synthase and suggest that this interaction may mechanistically influence metabolic flexibility. The topic is novel and of interest to this field. Though a considerable amount of work has been performed, some key experiments are missing which reduce the potential impact of the studies. Specific comments are listed below.

1) Though the comparison between the "MyoFibro gene signature" Fig. 1E, shares some similarity to the publicly available human heart failure patient tissue datasets (Fig. 1F&G), the separation between groups is much less pronounced. I realize that this is comparison between a single genetically-modified mouse model and a more complex human pathological condition which would likely offer a greater level of variability. Nevertheless, the suggestion that the human data "corroborates" that which is seen in the mouse model may be an overinterpretation. I would either soften the statement or add a qualifier to address this discrepancy. A similar comment could be applied to 2B relative to 2D&E.

2) In Fig. 3F, the Authors conclude that changes in mRNA expression (Pdk2) indicated a decrease in oxidative phosphorylation and ATP production in CST-KO heart and that CST can improve both endpoints. This may also be an overinterpretation. Changes in the mRNA would not necessarily provide definitive proof of this contention. These sentences should also be softened or a qualifier included.

3) From the results in 3G, H, & I, the authors conclude that CST increases insulin sensitivity in diabetic CST-KO heart as a result of AKT and GSK phosphorylation, but no measure of insulin sensitivity is included. This should be included to support the conclusions being drawn.

4) In general, the EMs in Fig. 5 are difficult to follow. SSM are alluded to but are not actually included in this figure. This figure should be reorganized to include EMs with both SSM and IFM so the reader can more easily follow the text narrative.

5) In many cases throughout the manuscript, the addition of CST to the CST-KO provides a hyper response as opposed to a restoration of a given endpoint. Fig. 5I & J are just one example, but others exist. Do the Authors have any idea as to why this is consistently observed and what it may biologically imply? An attempt to address this phenomenon would be appreciated. Further, in Fig. 5J, it is unclear why these specific mRNAs for the complexes were chosen. These are changes in select mRNA levels which may not reflect the protein content or more importantly, the content of the complex. Inclusion of protein complex contents (BN-PAGE) would be beneficial to complement these studies. The Authors should also consider whether CST is impacting respirasome formation.

6) The CST/F1-ATP synthase interaction studies are amongst the most exciting findings. The Authors utilize ligand affinity chromatography using heart homogenate. If the hypothesis is that CST binds to the ATP synthase, why not perform these studies on isolated mitochondria, which would provide a more rigorous analysis for testing the hypothesis? Determining if there was a difference in the binding dynamics of CST with SSM and IFM would be particularly relevant in light of the differential response of the both populations of mitochondria in Fig. 5.

7) Fig. 6I and 6J are alluded to in the text, but I do not see the panels. The structural studies that evaluate CST binding to the F1 ATP synthase lack evaluation of whether the binding of CST influences F1 ATP synthase functionality. The Authors speculate that abnormal physiological conditions influence the central axle rotation which would influence ATP synthesis (lines 656-659) but no attempt to prove this is provided. This is an important piece of data to include as it would provide an indication of the importance of the interaction in a functional context. Without this data it is unclear what the relevance is of CST binding to F1 ATP synthase.

8) Fig. 7G-L; as above, a BN-PAGE should be included for these studies to determine whether complexes are impacted as opposed to single constituents.

9) Fig. 7O; as in comment #7 above, does CST have an impact on complex V functionality or content?

Reviewer #2

(Remarks to the Author)
In this study, Chirasani et al. present a comprehensive investigation into the cardiometabolic functions of Catestatin (CST). Using CST knockout (CST-KO) mice, the authors demonstrate that CST supplementation reverses insulin resistance, enhances glucose metabolism, suppresses fatty acid uptake and oxidation, and increases mitochondrial ATP production. Notably, they identify ATP synthase as a direct CST binding target through affinity chromatography, supported by molecular docking and AlphaFold modeling. These results suggest a potential mechanistic link between CST and cardiac energetics. However, while the transcriptomics analyses are highly intriguing and reveal altered gene signatures associated with heart failure, they seem disconnected from the work on mitochondrial function. The specific mechanisms by which CST loss causes mitochondrial dysfunction, and how CST supplementation rescues the observed mitochondrial structural and functional defects, remain unclear. Clarifying these connections, providing direct evidence for changes in ATP synthase activity, and offering quantitative confirmation of key findings would significantly strengthen the conclusions.
Concerns
1. The finding that CST binds to ATP synthase are intriguing, but the impact of this interaction is unclear. The authors suggest that CST binging to ATP synthase enhances function and show increased membrane potential and total ATP levels in cardiomyocytes treated with the CST peptide. These measures do not necessarily mean that there is a direct effect on the ATP synthase itself. The authors should measure ATP synthase activity. Without a direct assessment, the claim that CST binding improves mitochondrial function through ATP synthase remains speculative.
2. Are there any other targets within the mitochondria at CST binds to?
3. Connection between CST and ceramide synthesis pathway is interesting and provides a potential pathway connecting CST loss to the mitochondrial dysfunction observed. Do the gene expression changes correlate to actual cardiac ceramide levels? Do ceramide levels increase in CST KO heart that is then reversed with CST supplementation?
4. The EM evidence of mitophagy in CST-KO hearts that could be rescued with CST is compelling, but qualitative. Addition of quantitative confirmation, quantification of mitophagy from the EM, or complementary confirmation of mitophagy by assessing mitophagy markers (eg. PINK1, Parkin, LC3-II) would strengthen the conclusions
5. In vitro experiments in the neonatal cardiomyocytes are elegant, but it is unclear why they were chosen for use. Neonatal cardiomyocytes are inherently immature (especially metabolically immature) vs. adult cardiomyocytes that may better reflect in vivo effects. Also, do the transcriptomics analyses also support a signature of elevated mitophagy?
6. The overall model presented needs more explanation—there are components included eg SIRT1 and PCG1a depicted in the model but not discussed as part of the results or in the discussion.
7. Is mitochondrial biogenesis increased with CST? Is this why there’s an increase in protein expression of OXPHOS subunits?
Minor concerns
1. CST dosing regimen should be included in the methods.
2. Why was the study only conducted on male mice? Would the same effects be seen in female mice?

Reviewer #3

(Remarks to the Author)
In this manuscript, Chirasani and his colleagues showed that CST supplementation in the hearts improves cardiac metabolism, thereby reversing insulin resistance. This manuscript contains a lot of data. However, several questions need to be fixed.
1. In CST-KO mice, both fatty acid metabolism and fatty acid uptake was activated in the cardiomyocytes. In contrast, fatty acid metabolism and fatty acid uptake showed a comparable decrease after CST supplementation. However, the number of LDs in the IFM images showed a dramatic decrease in CST-KO+CST group when compared with CST-KO+Sal, how could this happen?
2. The morphology of mitochondria in NRCVMs (Figure 7) is low-quantity and irregular. Moreover, the distribution of DAPI in the NRVCMs is quite puzzling. Please re-perform this experiments and replace the images.
3. The graphic abstract contains too many information which was not contained in this manuscript. For example, SIRT1, PGC1a, GABA…….. Please fix it.
4. CST supplementation promoted glucose metabolism while inhibited lipid metabolism in the hearts. The author further reported that CST improved mitochondrial function. Considering the critical role of mitochondria in both glucose oxidation and lipid oxidation, the improvement of mitochondrial function should also promote the lipid metabolism and thereby mitigating lipotoxicity in the heart. The author must provide more convincing reasons for how CST prevented lipotoxicity and

insulin resistance.
5. A seahorse mitochondrial respiration experiment should be performed to assess mitochondrial metabolism function with or without CST.
6. In figure4A, the author reported that CST reversed lipid-induced inhibition of glucose uptake in heart. In this part, PA was used to induce lipid overload and insulin resistance in the NRVCMs. Moreover, the author also showed that CST decreased the uptake of lipid. Considering the high demand of energy consumption of hearts, the increase of glucose uptake might be a compensation of decreased lipid oxidation, instead of improved insulin sensitivity.
7. Lipid oxidation serves as the major fuel in the normal heart. A decrease of FAs oxidation would lead to insufficient ATP production. The author should provide evidence that CST-induced metabolic reprogramming did not influence energy supply in the normal heart.
8. The entire manuscript needs to be structurally adjusted (eg. reduce unnecessary paragraphs) to enhance readability.

Version 1:

Reviewer comments:

Reviewer #1

(Remarks to the Author)
I appreciate the efforts undertaken by the Authors to address my critiques. For the most part, many of the critiques were addressed appropriately and in the case of some of the experimentation beyond the expertise of the research teams (i.e. BN-PAGE), I am fine with the responses. With that being said, one critique, which I think is easy to address, should still be considered. See below.

7) Reviewer comment: Fig. 6I and 6J are alluded to in the text, but I do not see the panels. The structural studies that evaluate CST binding to the F1 ATP synthase lack evaluation of whether the binding of CST influences F1 ATP synthase functionality. The Authors speculate that abnormal physiological conditions influence the central axle rotation which would influence ATP synthesis (lines 656-659) but no attempt to prove this is provided. This is an important piece of data to include as it would provide an indication of the importance of the interaction in a functional context. Without this data it is unclear what the relevance is of CST binding to F1 ATP synthase.
Response: We appreciate this important point. We performed ATP measurements with Seahorse (i.e. the response to Reviewer 3, comment 5) in H9c2 cells, but that there was no difference. Although CST markedly increased mitochondrial membrane potential ($\Delta\psi m$) (Fig. 8O) and ATP content (Fig. 8P) in neonatal primary cardiomyocytes, these effects were not recapitulated in H9c2 cells. This divergence is consistent with established metabolic differences between the two models. Neonatal cardiomyocytes possess a well-developed mitochondrial network, depend predominantly on oxidative phosphorylation, and exhibit strong spare respiratory capacity - conditions that allow CST to enhance $\Delta\psi m$ and ATP synthesis.
By contrast, H9c2 myoblast-derived cells rely primarily on glycolysis and display limited mitochondrial oxidative capacity, particularly when maintained in standard high-glucose media. Prior studies have shown that high-glucose H9c2 cells exhibit markedly reduced oxygen consumption, low respiratory control ratios, and increased lactate generation, confirming a predominantly glycolytic phenotype 62. Additional work demonstrates that undifferentiated H9c2 cells are metabolically immature and shift toward oxidative metabolism only upon differentiation. Moreover, comprehensive transcriptomic and functional profiling reveals that cardiac cell lines such as H9c2 have restricted metabolic responsiveness compared with primary cardiomyocytes. These metabolic constraints likely explain why CST-dependent increases in OCR or ATP production were not observed in H9c2 cells. Thus, CST's bioenergetic effects are model-dependent, with robust activity in metabolically competent primary cardiomyocytes but minimal impact in glycolytic, low-reserve H9c2 cells. Functional assays of ATP synthase activity would greatly strengthen the link between CST binding and metabolic regulation. As these studies also require specialized methodological capabilities, we plan to address ATP synthase functionality, including rotational catalysis, in future mechanistic work. We have added this clarification to the Discussion.

I appreciate the response, and I am in agreement with much of the comments that have been made by the Authors. Nevertheless, my suggestion for including a measure of ATP synthase functionality was so that the reader understood the importance of CST binding. The enzymatic evaluation of ATP synthase is fairly straightforward and is measured by following the decrease in NADH absorption at 340 nm, spectrophotometrically. This is just one reference (https://pubmed.ncbi.nlm.nih.gov/17092944/) but there are many. This can be performed on frozen samples, so it would not require repeating the study. I believe the inclusion of this data would strengthen the manuscript.

Reviewer #2

(Remarks to the Author)
In this revision, the authors have added a substantial amount of new data to address reviewer comments. Several aspects of the manuscript are strengthened. In particular, the new ceramide and triglyceride measurements provide convincing biochemical support for the transcriptomic findings and offer a plausible mechanistic link between CST loss, lipid accumulation, insulin resistance, and mitochondrial stress. The additional mitophagy analyses improve alignment between the structural EM observations and molecular readouts.
However, the primary concern regarding CST's effects on ATP synthase function remains unresolved. The interaction

between CST and ATP synthase is a central finding of this study, but the revised manuscript does not directly address whether CST alters ATP synthase activity. Seahorse-based measurements of OCR, including ATP-linked respiration and ATP rate assays, are indirect readouts of mitochondrial function and do not measure ATP synthase enzymatic activity. Similarly, increases in mitochondrial membrane potential or total cellular ATP content cannot be attributed specifically to modulation of ATP synthase without direct functional assessment of the enzyme.
The new Seahorse data are informative in that CST shows no effect on mitochondrial ATP production in H9c2 myoblast-derived cells, whereas increases in membrane potential and ATP content are observed in neonatal cardiomyocytes. The authors interpret this as a model- or bioenergetics-dependent effect of CST, which is a reasonable hypothesis. However, without direct measurements of ATP synthase activity (in both H9c2 myoblasts vs neonatal cardiomyocytes +/- CST), it is difficult to evaluate this interpretation. Thus, while the revision strengthens several parts of the study, the central mechanistic question regarding ATP synthase activity remains unanswered, and this limits the strength of the conclusions that can be drawn about CST's mode of action.

Reviewer #3

(Remarks to the Author)
The authors had addressed all my concerns in the revised manuscript.

Version 2:

Reviewer comments:

Reviewer #1

(Remarks to the Author)
No further comments.

Open Access This Peer Review File is licensed under a Creative Commons Attribution 4.0 International License, which permits use, sharing, adaptation, distribution and reproduction in any medium or format, as long as you give appropriate credit to the original author(s) and the source, provide a link to the Creative Commons license, and indicate if changes were made.
In cases where reviewers are anonymous, credit should be given to 'Anonymous Referee' and the source.
The images or other third party material in this Peer Review File are included in the article's Creative Commons license, unless indicated otherwise in a credit line to the material. If material is not included in the article's Creative Commons license and your intended use is not permitted by statutory regulation or exceeds the permitted use, you will need to obtain permission directly from the copyright holder.
To view a copy of this license, visit https://creativecommons.org/licenses/by/4.0/

RESPONSE TO REVIEWERS' COMMENTS

We thank all reviewers for their careful evaluation of our manuscript and for bringing their deep expertise to the critique. We also appreciate their encouraging remarks, including descriptions such as *"novel and of interest to this field", and "exciting findings"* (reviewer 1); *"comprehensive investigation", "intriguing", and "interesting"* (reviewer 2); and *"lot of data"* (reviewer 3). All reviewers identified areas that required strengthening. We have addressed each point in detail and performed additional experiments to reinforce the rigor and clarity of our study. A summary of the major revisions is provided below.

Major changes:

1. **Glucose homeostasis:** We performed a glucose tolerance test (GTT), which demonstrated significantly improved glucose excursion in CST-supplemented CST-KO mice (**new Fig. 4A–C**).
2. **Hepatic glucose output:** A pyruvate tolerance test (PTT) revealed reduced hepatic glucose production in CST-treated CST-KO mice (**new Fig. 4D–F**).
3. **Insulin sensitivity:** We completed an insulin tolerance test (ITT), which showed a more pronounced hypoglycemic response in CST-administered CST-KO mice, confirming enhanced whole-body insulin sensitivity (**new Fig. 4G–I**).
4. **Ultrastructural analysis:** We added new transmission electron micrographs illustrating sub-sarcolemmal mitochondrial architecture and morphological abnormalities in CST-KO hearts, together with CST-mediated rescue (**new Fig. 6A–H, Q, R, V&W**).
5. **Lipid metabolism:** Additional biochemical analyses revealed elevated triglyceride and ceramide levels in CST-KO hearts, both of which were normalized by CST supplementation (**new Fig. 5E&F**).
6. **Mitochondrial biogenesis:** We incorporated new RT-qPCR data showing CST-induced upregulation of ***Sirt1*** and ***Pgc1a***, supporting CST's role in enhancing mitochondrial biogenesis (**new Fig. 8N**).
7. **Mitophagy markers:** We performed RT-qPCR analysis of canonical mitophagy regulators (*PINK1**,** PRKN/Parkin**,** MAP1LC3B*). CST-KO hearts exhibited marked upregulation of all three genes, whereas CST supplementation restored expression toward WT levels, indicating that CST loss activates the *PINK1 → PRKN (Park2*) *→ MAP1LC3B* mitophagy pathway and that CST replacement attenuates this mitochondrial stress response (**new Fig. 6U**). These molecular findings provide quantitative confirmation that complements our EM evidence of mitophagy.
8. **Transcriptomic validation of mitophagy:** Bulk RNA sequencing revealed significant enrichment of the "positive regulation of mitophagy" GO gene set in CST-KO hearts, accompanied by prominent upregulation of *PRKN (Park2)* and an elevated mitophagy gene-set score. CST supplementation reversed these transcriptomic signatures, reducing PRKN pathway activity and the overall mitophagy score (**new S-Fig. 1A-D**). These data independently validate that CST deficiency activates cardiac mitophagy, consistent with our EM (**new Fig. 6W&Y**) and RT-qPCR results (**new Fig. 6U)**.
9. **Localization of CST:** We added new immunohistochemical analyses demonstrating co-localization of endogenous CST with mitochondria, further supporting a functional relationship between CST and mitochondrial regulation (**new Fig. 8A-G**).
10. **Mitochondrial energetics:** We performed the Seahorse XF Cell Mito Stress Test in H9c2 cells. Across multiple CST doses, CST did not significantly alter basal oxygen consumption (**S-Fig. 7A**), non-mitochondrial respiration (**S-Fig. 7B**), basal respiration (**S-Fig. 7C**), proton leak (**S-Fig. 7D**), ATP-linked respiration (**S-Fig. 7E**), maximal respiration (**S-Fig. 7F**), or spare respiratory capacity (**S-Fig. 7G**). The Seahorse ATP Rate Assay similarly showed no CST-dependent changes in glycolytic ATP rate (**S-Fig. 7H**), mitochondrial ATP rate (**S-Fig. 7I**), or total ATP rate (**S-Fig. 7J**). These results have been incorporated into the revised manuscript.
11. **Structural mechanism of CST–ATP synthase interaction:** To strengthen the mechanistic insights regarding how CST binding alters ATP synthase function, we performed *de novo*

comparative molecular dynamics (MD) simulations of CST-bound versus unbound ATP synthase. These analyses revealed reduced atomic fluctuations at the d–c–f interface and increased flexibility at the opposing a–e–b interface upon CST binding (**new Fig. 7G**). Angular displacement analysis further demonstrated decreased tilt of the d–c–f subunits and increased tilt of the a–e–b subunits relative to the central axle (**new Fig. 7H**). Together, these CST-induced conformational shifts support prior mechanistic models proposing that alternating subunit displacements facilitate ADP binding and ATP release. The addition of these simulations provides a structural framework that unifies our biochemical, ultrastructural, and functional findings, and offers a mechanistic explanation for CST-mediated modulation of mitochondrial ATP synthase.

Point-by-point response to reviewers' comments

Response to Reviewer #1:

We thank this reviewer for their thoughtful evaluation of our manuscript and for recognizing the novelty and significance of our work.

1) Reviewer comment: Though the comparison between the "MyoFibro gene signature" Fig. 1E, shares some similarity to the publicly available human heart failure patient tissue datasets (Fig. 1F&G), the separation between groups is much less pronounced. I realize that this is comparison between a single genetically-modified mouse model and a more complex human pathological condition which would likely offer a greater level of variability. Nevertheless, the suggestion that the human data "corroborates" that which is seen in the mouse model may be an overinterpretation. I would either soften the statement or add a qualifier to address this discrepancy. A similar comment could be applied to 2B relative to 2D&E.

Response: We appreciate this insightful comment. As suggested, we have revised our wording throughout the manuscript to soften the language and avoid overinterpretation. In addition, we expanded our analysis to include a broader set of publicly available human, mouse, and rat transcriptomic datasets to demonstrate the robustness of our findings across species. The revised figure panels now include:

- **Fig. 1H:** Human (GSE302337), mouse (GSE133054, GSE241771, GSE298801, GSE267693, GSE234210), and rat (GSE144548) datasets
- **Fig. 2F:** Human (GSE302337), mouse (GSE241771, GSE267693), and rat (GSE144548, GSE240921) datasets
- **Fig. 2J:** Human (GSE302337), mouse (GSE241771, GSE267693), and rat (GSE144548, GSE240921) datasets

These additions strengthen cross-species consistency while maintaining the interpretational caution recommended by the reviewer.

2) Reviewer comment: In Fig. 3F, the Authors conclude that changes in mRNA expression (Pdk2) indicated a decrease in oxidative phosphorylation and ATP production in CST-KO heart and that CST can improve both endpoints. This may also be an overinterpretation. Changes in the mRNA would not necessarily provide definitive proof of this contention. These sentences should also be softened or a qualifier included.

Response: We fully agree. We have revised the relevant statements to a more measured interpretation, emphasizing that decreased *Pdk2* expression is *consistent with*, but does not by itself prove, enhanced ATP production.

3) Reviewer comment: From the results in 3G, H, & I, the authors conclude that CST increases insulin sensitivity in diabetic CST-KO heart as a result of AKT and GSK phosphorylation, but no measure of insulin sensitivity is included. This should be included to support the conclusions being drawn.

Response: We thank the reviewer for this important suggestion. To directly assess insulin sensitivity, we performed three additional experiments, now included as **new Fig. 4A–I**:

1. **Glucose tolerance test (GTT):** CST supplementation improved glucose excursion in CST-KO mice (**new Fig. 4A–C**).
2. **Pyruvate tolerance test (PTT):** CST treatment reduced hepatic glucose output (**new Fig. 4D–F**).
3. **Insulin tolerance test (ITT):** CST-supplemented CST-KO mice exhibited pronounced hypoglycemia (**new Fig. 4G–I**).

Together, these data demonstrate that CST functions as an insulin-sensitizing peptide and reverses metabolic inflexibility in CST-KO hearts.

4) Reviewer comment: In general, the EMs in Fig. 5 are difficult to follow. SSM are alluded to but are not actually included in this figure. This figure should be reorganized to include EMs with both SSM and IFM so the reader can more easily follow the text narrative.

Response: We appreciate this recommendation. We have reorganized and updated the figure to include both subsarcolemmal (SSM) and interfibrillar (IFM) mitochondria in **revised Fig. 6**, enabling clearer alignment between text and images.

5) Reviewer comment: In many cases throughout the manuscript, the addition of CST to the CST-KO provides a hyperresponse as opposed to a restoration of a given endpoint. Fig. 5I & J are just one example, but others exist. Do the Authors have any idea as to why this is consistently observed and what it may biologically imply? An attempt to address this phenomenon would be appreciated. Further, in Fig.5J, it is unclear why these specific mRNAs for the complexes were chosen. These are changes in select mRNA levels which may not reflect the protein content or more importantly, the content of the complex. Inclusion of protein complex contents (BN-PAGE) would be beneficial to complement these studies. The Authors should also consider whether CST is impacting respirasome formation.

Response: We thank the reviewer for highlighting this recurring pattern. To aid interpretation, we have summarized the fold-changes across metabolic, signaling, and structural readouts (data listed in the response). In most cases, CST supplementation restores CST-KO values to near-WT levels (e.g., glycogen content, FA utilization, mitochondrial ultrastructure).

Where apparent "hyperresponses" occur (e.g., AKT/GSK3β phosphorylation and select contractile genes), we propose that CST may exert compensatory upregulation in CST-deficient tissues with chronic metabolic stress. We have added this explanation to the Discussion.

Regarding BN-PAGE: we agree that assessing respirasome and complex abundance would significantly strengthen the mechanistic interpretation. However, our laboratory does not currently have the expertise to perform BN-PAGE. We have acknowledged this limitation and will address it comprehensively in future mechanistic studies.

Fig. 3E: 2DG uptake: WT+Sal: 6.016 vs CST-KO+CST: 8.28 = ~1.37-fold;
G6P utilization: WT+Sal: 5.593 vs CST-KO+CST: 7.09 = ~1.27%.

Fig. 3F: Slc2a4: WT+Sal: 1 vs CST-KO+CST: ~3.42-fold;
Pfkm: WT+Sal: 0.775 vs CST-KO+CST: 1.84: ~2.37-fold;
Pdk2: WT+Sal: 0.854 vs CST-KO+CST: 0.86 = ~1.006-fold.

Fig. 3Q: Glycogen content: WT-Fed+Sal: 6.596 vs CST-KO-Fed+CST: 6.563 = ~0.99-fold;
Fig. 3P: Glycogen granules: WT-Fed+Sal: 111 vs CST-KO-Fed+CST: 108 = 0.97-fold
Fig. 3H: AKT: WT+Sal: 0.4 vs CST-KO+CST: 0.956 = 2.39-fold
Fig. 3I: GSK-3b: WT+Sal: 0.426 vs CST-KO+CST: 1.183 = 2.77-fold
Fig. 4B: FA uptake: WT+Sal: 9.84 vs CST-KO+CST: 13.45 = 1.367-fold
Fig. 4C: FA utilization: WT+Sal: 5.128 vs CST-KO+CST: 6.09 = 1.187-fold

Fig. 4D: *Cd36*: WT+Sal: 1 vs CST-KO+CST: 1.156 = 1.156-fold

Slc27a1: WT+Sal: 1 vs CST-KO+CST: 1.269 = 1.269-fold
Slc27a2: WT+Sal: 1 vs CST-KO+CST: 1.299 = 1.299-fold
Dgat2: WT+Sal: 1 vs CST-KO+CST: 0.936 = 0.936-fold
Sptlc1: WT+Sal: 1 vs CST-KO+CST: 1.120 = 1.120-fold
Cers2: WT+Sal: 1 vs CST-KO+CST: 1.699 = 1.699-fold
Ppara: WT+Sal: 1 vs CST-KO+CST: 1.368 = 1.368-fold

S-Fig. 1: ***Tnni3***: WT+Sal: 1 vs CST-KO+CST: 3.868 = 3.868-fold
Tnnt2: WT+Sal: 1 vs CST-KO+CST: 3.194 = 3.194-fold
Tpm: WT+Sal: 1 vs CST-KO+CST: 1.122 = 1.122-fold
Ttn: WT+Sal: 1 vs CST-KO+CST: 1.806 = 1.806-fold
Actc1: WT+Sal: 1 vs CST-KO+CST: 3.877 = 3.877-fold
Myl4: WT+Sal: 1 vs CST-KO+CST: 3.771 = 3.771-fold
Myl7: WT+Sal: 1 vs CST-KO+CST: 4.301 = 4.301-fold

Fig. 6A&G: **SSM sarcomere length**: WT+Sal: 2.087 vs CST-KO+CST: 2.215 = 1.061-fold
Fig. 6B&H: SSM Cristae surface area: WT+Sal: 34.108 vs CST-KO+CST: 32.693 = 0.958-fold
Fig. 6I&O: IFM sarcomere length: WT+Sal: 2.275 vs CST-KO+CST: 2.326 = 1.022-fold
Fig. 6J&P: IFM Cristae surface area: WT+Sal: 32.87 vs CST-KO+CST: 36.48 = 1.11-fold

6) Reviewer comment: The CST/F1-ATP synthase interaction studies are amongst the most exciting findings. The Authors utilize ligand affinity chromatography using heart homogenate. If the hypothesis is that CST binds to the ATP synthase, why not perform these studies on isolated mitochondria, which would provide a more rigorous analysis for testing the hypothesis? Determining if there was a difference in the binding dynamics of CST with SSM and IFM would be particularly relevant in light of the differential response of the both populations of mitochondria in Fig. 5.

Response: We agree that isolating mitochondria and performing fraction-specific binding studies would provide a rigorous test of our hypothesis. Because these experiments require advanced technical expertise and specialized instrumentation, they are beyond the current scope of this study. We fully intend to incorporate these analyses in a dedicated follow-up manuscript.

7) Reviewer comment: Fig. 6I and 6J are alluded to in the text, but I do not see the panels. The structural studies that evaluate CST binding to the F1 ATP synthase lack evaluation of whether the binding of CST influences F1 ATP synthase functionality. The Authors speculate that abnormal physiological conditions influence the central axle rotation which would influence ATP synthesis (lines 656-659) but no attempt to prove this is provided. This is an important piece of data to include as it would provide an indication of the importance of the interaction in a functional context. Without this data it is unclear what the relevance is of CST binding to F1 ATP synthase.
Response: We appreciate this important point. We performed ATP measurements with Seahorse (i.e. the response to Reviewer 3, comment 5) in H9c2 cells, but that there was no difference. Although CST markedly increased mitochondrial membrane potential (Δψm) (**Fig. 8O**) and ATP content (**Fig. 8P**) in neonatal primary cardiomyocytes, these effects were not recapitulated in H9c2 cells. This divergence is consistent with established metabolic differences between the two models. Neonatal cardiomyocytes possess a well-developed mitochondrial network, depend predominantly on oxidative phosphorylation, and exhibit strong spare respiratory capacity - conditions that allow CST to enhance Δψm and ATP synthesis.

By contrast, H9c2 myoblast-derived cells rely primarily on glycolysis and display limited mitochondrial oxidative capacity, particularly when maintained in standard high-glucose media. Prior studies have shown that high-glucose H9c2 cells exhibit markedly reduced oxygen consumption, low respiratory control ratios, and increased lactate generation, confirming a predominantly glycolytic phenotype [62]. Additional work demonstrates that undifferentiated H9c2 cells are metabolically immature and shift toward oxidative metabolism only upon differentiation. Moreover, comprehensive

transcriptomic and functional profiling reveals that cardiac cell lines such as H9c2 have restricted metabolic responsiveness compared with primary cardiomyocytes. These metabolic constraints likely explain why CST-dependent increases in OCR or ATP production were not observed in H9c2 cells. Thus, CST's bioenergetic effects are model-dependent, with robust activity in metabolically competent primary cardiomyocytes but minimal impact in glycolytic, low-reserve H9c2 cells. Functional assays of ATP synthase activity would greatly strengthen the link between CST binding and metabolic regulation. As these studies also require specialized methodological capabilities, we plan to address ATP synthase functionality, including rotational catalysis, in future mechanistic work. We have added this clarification to the Discussion.

8) Reviewer comment: Fig. 7G-L; as above, a BN-PAGE should be included for these studies to determine whether complexes are impacted as opposed to single constituents.

Response: We agree that BN-PAGE analyses would be highly informative. Since our group does not currently have expertise in this technique, we will include BN-PAGE–based evaluation of respiratory supercomplexes in future follow-up studies.

9) Reviewer comment: Fig. 7O; as in comment #7 above, does CST have an impact on complex V functionality or content?

Response: We appreciate the reviewer's interest in this mechanistic question. We have noted this as an important future direction and will address CST-dependent regulation of complex V content and functionality in upcoming studies focused specifically on mitochondrial bioenergetics.

Response to Reviewer #2:

We thank this reviewer for the thoughtful assessment of our manuscript and for highlighting the valuable connections between CST signaling, mitochondrial biology, and transcriptomic remodeling. We appreciate the constructive suggestions, which have helped us strengthen the clarity and rigor of the revised manuscript.

General Response

Reviewer comment: In this study, Chirasani et al. present a comprehensive investigation into the cardiometabolic functions of Catestatin (CST). Using CST knockout (CST-KO) mice, the authors demonstrate that CST supplementation reverses insulin resistance, enhances glucose metabolism, suppresses fatty acid uptake and oxidation, and increases mitochondrial ATP production. Notably, they identify ATP synthase as a direct CST binding target through affinity chromatography, supported by molecular docking and AlphaFold modeling. These results suggest a potential mechanistic link between CST and cardiac energetics. However, while the transcriptomics analyses are highly intriguing and reveal altered gene signatures associated with heart failure, they seem disconnected from the work on mitochondrial function. The specific mechanisms by which CST loss causes mitochondrial dysfunction, and how CST supplementation rescues the observed mitochondrial structural and functional defects, remain unclear. Clarifying these connections, providing direct evidence for changes in ATP synthase activity, and offering quantitative confirmation of key findings would significantly strengthen the conclusions.

Response: We appreciate this important point. We performed ATP measurements with Seahorse (i.e. the response to Reviewer 3, comment 5) in H9c2 cells, but that there was no difference (**new Fig. S-6J**). Although CST markedly increased mitochondrial membrane potential (Δψm) (**Fig. 8O**) and ATP content (**Fig. 8P**) in neonatal primary cardiomyocytes, these effects were not recapitulated in H9c2 cells. This divergence is consistent with established metabolic differences between the two models. Neonatal cardiomyocytes possess a well-developed mitochondrial network, depend predominantly on oxidative phosphorylation, and exhibit strong spare respiratory capacity - conditions that allow CST to enhance Δψm and ATP synthesis.

By contrast, H9c2 myoblast-derived cells rely primarily on glycolysis and display limited mitochondrial oxidative capacity, particularly when maintained in standard high-glucose media. Prior studies have shown that high-glucose H9c2 cells exhibit markedly reduced oxygen consumption, low respiratory control ratios, and increased lactate generation, confirming a predominantly glycolytic phenotype [62]. Additional work demonstrates that undifferentiated H9c2 cells are metabolically immature and shift toward oxidative metabolism only upon differentiation. Moreover, comprehensive transcriptomic and functional profiling reveals that cardiac cell lines such as H9c2 have restricted metabolic responsiveness compared with primary cardiomyocytes. These metabolic constraints likely explain why CST-dependent increases in OCR or ATP production were not observed in H9c2 cells. Thus, CST's bioenergetic effects are model-dependent, with robust activity in metabolically competent primary cardiomyocytes but minimal impact in glycolytic, low-reserve H9c2 cells. We agree that directly assessing ATP synthase activity would further strengthen our mechanistic conclusions. These experiments require methodological optimization and will be part of a dedicated follow-up study, where we will also investigate mitochondrial target specificity and interaction dynamics of CST.

Point-by-point responses

1. Reviewer comment: The finding that CST binds to ATP synthase are intriguing, but the impact of this interaction is unclear. The authors suggest that CST binging to ATP synthase enhances function and show increased membrane potential and total ATP levels in cardiomyocytes treated with the CST peptide. These measures do not necessarily mean that there is a direct effect on the ATP synthase itself. The authors should measure ATP synthase activity. Without a direct assessment, the claim that CST binding improves mitochondrial function through ATP synthase remains speculative.

Response: Please see our reply to the general comment above.

2. Reviewer comment: Are there any other targets within the mitochondria at CST binds to?

Response: At present, we do not know whether CST interacts with additional mitochondrial targets. We will address this important question through proteomic pull-down studies and mitochondrial interactome profiling in a separate follow-up project.

3. Reviewer comment: Connection between CST and ceramide synthesis pathway is interesting and provides a potential pathway connecting CST loss to the mitochondrial dysfunction observed. Do the gene expression changes correlate to actual cardiac ceramide levels? Do ceramide levels increase in CST KO heart that is then reversed with CST supplementation?

Response: We thank the reviewer for this excellent question. We have now quantified triglycerides and ceramides in left ventricular tissues. CST-KO mice exhibit a ~1.5-fold increase in triglycerides (**new Fig. 5E**) and a ~1.7-fold increase in ceramide levels (**Fig. 5F**). Both metabolic abnormalities are restored to WT levels following CST supplementation. These results provide biochemical confirmation that aligns with our transcriptomic observations linking CST deficiency to ceramide dysregulation.

4. Reviewer comment: The EM evidence of mitophagy in CST-KO hearts that could be rescued with CST is compelling, but qualitative. Addition of quantitative confirmation, quantification of mitophagy from the EM, or complementary confirmation of mitophagy by assessing mitophagy markers (eg. PINK1, Parkin, LC3-II) would strengthen the conclusions.

Response: We thank the reviewer for this important suggestion. In addition to revising the EM panel to include representative images, we performed qRT-PCR analyses of canonical mitophagy markers, which demonstrated significant upregulation of *PINK1*, *PRKN* (*Parkin*), and *MAP1LC3B* in CST-KO hearts. Importantly, expression of all three genes returned toward WT levels following CST supplementation**,** indicating that CST loss activates the *PINK1* → *PRKN* (Parkin) → *MAP1LC3B* mitophagy program, whereas CST replacement mitigates this mitochondrial stress response (**new S-Fig. 1**). These molecular data provide independent quantitative confirmation that complements our EM findings.

5. In vitro experiments in the neonatal cardiomyocytes are elegant, but it is unclear why they were chosen for use. Neonatal cardiomyocytes are inherently immature (especially metabolically immature) vs. adult cardiomyocytes that may better reflect in vivo effects. Also, do the transcriptomics analyses also support a signature of elevated mitophagy?

Response: We used neonatal cardiomyocytes because they remain the standard, well-validated model for mechanistic studies requiring mitochondrial assays, pharmacological perturbations, peptide uptake, and transfection-based manipulation. They provide high reproducibility and technical robustness, enabling detailed dissection of CST-mediated mitochondrial mechanisms. We agree that neonatal cardiomyocytes are metabolically less mature than adult myocytes, and we will incorporate adult cardiomyocytes in future mechanistic studies to further validate our findings.

Transcriptomics analyses: To determine whether CST regulates mitochondrial quality control pathways in vivo, we analyzed mitophagy-associated transcripts using bulk RNA sequencing. CST-KO hearts exhibited a significant enrichment of the "positive regulation of mitophagy" GO gene set, including prominent upregulation of *PRKN* (*Park2*) and an elevated global mitophagy gene-set score relative to WT. CST supplementation reversed these transcriptomic changes, reducing both PRKN pathway activity and the overall mitophagy score (**new S-Fig. 1**). These transcriptomic data provide independent confirmation that CST deficiency activates cardiac mitophagy, consistent with our EM and qRT-PCR findings.

6. Reviewer comment: The overall model presented needs more explanation—there are components included eg SIRT and PCG1a depicted in the model but not discussed as part of the results or in the discussion.

Response: We thank the reviewer for pointing this out. Our new RT-qPCR data show that CST supplementation increases expression of *Sirt1* and *Pgc1a* genes (**new Fig. 8N**), two central regulators of mitochondrial biogenesis and oxidative metabolism. These results are now described in both the Results and Discussion sections, and the model has been revised for consistency.

7. Reviewer comment: Is mitochondrial biogenesis increased with CST? Is this why there's an increase in protein expression of OXPHOS subunits?

Response: Based on CST-induced upregulation of *Sirt1* and *Pgc1a* (**new Fig. 8N**), it is reasonable to infer that CST promotes mitochondrial biogenesis. This may partially account for the observed increase in OXPHOS protein expression. We have clarified this interpretation in the Discussion.

Minor concerns

1. Reviewer comment: CST dosing regimen should be included in the methods.

Response: As suggested, the CST dosing regimen is now fully described in the Methods section.

2. Reviewer comment: Why was the study only conducted on male mice? Would the same effects be seen in female mice?

Response: Metabolic studies examining insulin resistance, glucose tolerance, and lipid handling are typically performed in male mice to avoid confounding variability from estrous-cycle–dependent metabolic fluctuations. Importantly, our recent publication (https://doi.org/10.1038/s42003-025-09135-z) demonstrates that CST-mediated suppression of gluconeogenesis is actually more pronounced in females. We therefore plan to include both sexes in future mitochondrial and metabolic investigations.

Response to Reviewer #3:

We thank Reviewer #3 for recognizing the depth of the dataset presented in our study and for providing constructive feedback that greatly enhanced the clarity, rigor, and overall quality of the revised manuscript.

1. Reviewer comment: In CST-KO mice, both fatty acid metabolism and fatty acid uptake was activated in the cardiomyocytes. In contrast, fatty acid metabolism and fatty acid uptake showed a comparable decrease after CST supplementation. However, the number of LDs in the IFM images showed a dramatic decrease in CST-KO+CST group when compared with CST-KO+Sal, how could this happen?

Response: We thank the reviewer for this important observation. In the original submission, two EM images displaying the strongest CST effect were inadvertently selected. These were not representative of the average phenotype. We have now replaced them with representative images (**new Fig. 6K, O**) that more accurately reflect the biochemical data, thereby improving concordance between ultrastructural and metabolic findings.

2. Reviewer comment: The morphology of mitochondria in NRCVMs (Figure 7) is low-quantity and irregular. Moreover, the distribution of DAPI in the NRVCMs is quite puzzling. Please re-perform this experiment and replace the images.

Response: As recommended, we repeated the experiment using H9c2 cardiomyoblasts, which provide more uniform cellular morphology and yield highly reproducible mitochondrial imaging. New high-quality images are now included in **Revised Fig. 8A-D** along with quantification (**new Fig. 8E-G**).

3. Reviewer comment: The graphic abstract contains too many information which was not contained in this manuscript. For example, SIRT1, PGC1a, GABA…….. Please fix it.

Response: We appreciate this helpful suggestion. The graphic abstract has been revised by removing ERRα and GABA. In addition, new RT-qPCR data demonstrating CST-induced upregulation of *Sirt1* and *Pgc1a* have been incorporated into both the Results and Discussion. The updated graphic abstract now accurately reflects the revised manuscript content.

4. Reviewer comment: CST supplementation promoted glucose metabolism while inhibited lipid metabolism in the hearts. The author further reported that CST improved mitochondrial function. Considering the critical role of mitochondria in both glucose oxidation and lipid oxidation, the improvement of mitochondrial function should also promote the lipid metabolism and thereby mitigating lipotoxicity in the heart. The author must provide more convincing reasons for how CST prevented lipotoxicity and insulin resistance.

Response: We thank the reviewer for raising this key mechanistic question. CST regulates systemic and cardiac lipid homeostasis through multiple complementary pathways. Prior work demonstrates that CST enhances adipose lipolysis, increases hepatic fatty-acid oxidation, and reduces lipid re-esterification by suppressing α-adrenergic signaling and augmenting leptin-receptor pathways (PMID: 22535963). CST also improves whole-body insulin sensitivity by reducing inflammation, suppressing gluconeogenesis, limiting macrophage infiltration, and alleviating ER stress (PMID: 29432123; PMID: 32180905). In the present study, CST reduced cardiac triglyceride (**new Fig. 5E**) and ceramide accumulation (**new Fig. 5F**) - two major mediators of lipotoxicity and insulin resistance. These combined actions redirect lipid flux away from pathological storage and toward oxidative disposal, thereby mitigating lipotoxic stress in CST-KO hearts. We have elaborated this mechanism in the revised Discussion.

5. Reviewer comment: A seahorse mitochondrial respiration experiment should be performed to assess mitochondrial metabolism function with or without CST.

Response: In response, we performed the Seahorse XF Cell Mito Stress Test in H9c2 cells. Across multiple CST doses, no significant changes were observed in basal oxygen consumption (**S-Fig. 7A**), non-mitochondrial respiration (**S-Fig. 7B**), basal respiration (**S-Fig. 7C**), proton leak (**S-Fig. 7D**), ATP-linked respiration (**S-Fig. 7E**), maximal respiration (**S-Fig. 7F**), or spare respiratory capacity (**S-Fig. 7G**). The Seahorse ATP Rate Assay similarly showed no significant CST-dependent changes in

glycolytic ATP rate (**S-Fig. 7H**), mitochondrial ATP rate (**S-Fig. 7I**), or total ATP rate (**S-Fig. 7J**). These results have been added to the revised manuscript.

Although CST markedly increased mitochondrial membrane potential (Δψm) (**Fig. 8O**) and ATP content (**Fig. 8P**) in neonatal primary cardiomyocytes, these effects were not recapitulated in H9c2 cells. This divergence is consistent with established metabolic differences between the two models. Neonatal cardiomyocytes possess a well-developed mitochondrial network, depend predominantly on oxidative phosphorylation, and exhibit strong spare respiratory capacity - conditions that allow CST to enhance Δψm and ATP synthesis.

By contrast, H9c2 myoblast-derived cells rely primarily on glycolysis and display limited mitochondrial oxidative capacity, particularly when maintained in standard high-glucose media. Prior studies have shown that high-glucose H9c2 cells exhibit markedly reduced oxygen consumption, low respiratory control ratios, and increased lactate generation, confirming a predominantly glycolytic phenotype [62]. Additional work demonstrates that undifferentiated H9c2 cells are metabolically immature and shift toward oxidative metabolism only upon differentiation. Moreover, comprehensive transcriptomic and functional profiling reveals that cardiac cell lines such as H9c2 have restricted metabolic responsiveness compared with primary cardiomyocytes. These metabolic constraints likely explain why CST-dependent increases in OCR or ATP production were not observed in H9c2 cells. Thus, CST's bioenergetic effects are model-dependent, with robust activity in metabolically competent primary cardiomyocytes but minimal impact in glycolytic, low-reserve H9c2 cells.

6. Reviewer comment: In figure 4A, the author reported that CST reversed lipid-induced inhibition of glucose uptake in heart. In this part, PA was used to induce lipid overload and insulin resistance in the NRVCMs. Moreover, the author also showed that CST decreased the uptake of lipid. Considering the high demand of energy consumption of hearts, the increase of glucose uptake might be a compensation of decreased lipid oxidation, instead of improved insulin sensitivity.

Response: We appreciate this insightful interpretation. To distinguish between metabolic compensation and true improvement in insulin sensitivity, we performed in vivo metabolic testing in CST-KO mice supplemented with CST. CST significantly improved glucose tolerance (as shown by GTT; **new Fig. 4A-C**), reduced hepatic glucose output (as shown by PTT; **new Fig. 4D-F**), and enhanced whole-body insulin responsiveness (as shown by ITT; **new Fig. 4G-I**). These systemic results, combined with CST-mediated reversal of lipid-induced suppression of glucose uptake in cardiomyocytes, indicate that CST restores insulin signaling rather than merely compensating for reduced FA oxidation. This clarification has been incorporated into the revised text.

7. Reviewer comment: Lipid oxidation serves as the major fuel in the normal heart. A decrease of FAs oxidation would lead to insufficient ATP production. The author should provide evidence that CST-induced metabolic reprogramming did not influence energy supply in the normal heart.

Response: We thank the reviewer for this important point. CST-treated CST-KO hearts maintain normal ATP content and mitochondrial membrane potential despite a modest reduction in FA oxidation. This preservation of energetic homeostasis likely reflects CST-mediated enhancement of glucose oxidation, improved insulin signaling, strengthened mitochondrial quality control pathways, and increased expression of mitochondrial biogenesis regulators (*Sirt1*, *Pgc1a;* **new Fig. 8N**). These mechanisms collectively ensure sufficient ATP generation in CST-supplemented hearts even with partial substrate rebalancing. We have clarified this mechanistic rationale in the revised Discussion.

8. Reviewer comment: The entire manuscript needs to be structurally adjusted (eg. reduce unnecessary paragraphs) to enhance readability.

Response: We appreciate this recommendation. The manuscript has undergone substantial restructuring, including removal of redundant text, refinement of figure - text integration, and improved logical flow. These revisions have significantly enhanced readability and coherence.

RESPONSE TO REVIEWERS' COMMENTS

We thank all reviewers for their careful evaluation of our revised manuscript. We are pleased that Reviewer #3 is satisfied with our responses. Reviewers #1 and #2 requested direct assessment of ATP synthase activity to strengthen the mechanistic link between CST and mitochondrial bioenergetics. In response, we have performed new ATP synthase (Complex V) activity assays and incorporated these data into the revised manuscript (Fig. 8P–Q). Below we provide a point-by-point response:

Point-by-point response to reviewers' comments

Response to Reviewer #1:

We thank this reviewer for the thoughtful and constructive evaluation of our manuscript.

Reviewer comment: I appreciate the response, and I am in agreement with much of the comments that have been made by the Authors. Nevertheless, my suggestion for including a measure of ATP synthase functionality was so that the reader understood the importance of CST binding. The enzymatic evaluation of ATP synthase is fairly straightforward and is measured by following the decrease in NADH absorption at 340 nm, spectrophotometrically. This is just one reference (https://pubmed.ncbi.nlm.nih.gov/17092944/) but there are many. This can be performed on frozen samples, so it would not require repeating the study. I believe the inclusion of this data would strengthen the manuscript.

Response: We appreciate the reviewer's insightful suggestion. In accordance with this recommendation, we performed direct ATP synthase (Complex V) activity assays using a spectrophotometric NADH oxidation–based method, monitoring the decrease in absorbance at 340 nm.

In the revised Results section, we now state:

"To determine whether CST enhances mitochondrial function, we assessed mitochondrial membrane potential (Δψm) in neonatal mouse cardiomyocytes; ATP synthase (Complex V) activity H9C2 cardiomyocytes, and total cellular ATP levels in neonatal mouse cardiomyocytes. CST treatment significantly increased mitochondrial membrane potential compared with PBS-treated controls (**Fig. 8O**), indicating enhanced electrochemical gradient integrity. Consistent with this observation, mitochondria isolated from CST-treated cells exhibited increased ATP synthase catalytic activity. Kinetic analysis demonstrated a greater rate of NADH oxidation in CST-treated mitochondria (**Fig. 8P**), and quantitative analysis confirmed a significant elevation in Complex V activity (**Fig. 8Q**). Importantly, the enhancement in mitochondrial enzymatic function translated into increased intracellular ATP levels following 24 h CST treatment (**Fig. 8R**). Together, these findings demonstrate that CST augments mitochondrial bioenergetic capacity by strengthening membrane potential, increasing ATP synthase activity, and elevating ATP production in cardiomyocytes."

These newly generated data directly address the reviewer's concern and strengthen the mechanistic connection between CST treatment and enhanced mitochondrial ATP synthase activity.

Response to Reviewer #2:

We thank this reviewer for the careful assessment and constructive comments.

Reviewer comment: The new Seahorse data are informative in that CST shows no effect on mitochondrial ATP production in H9c2 myoblast-derived cells, whereas increases in membrane potential and ATP content are observed in neonatal cardiomyocytes. The authors interpret this as a model- or bioenergetics-dependent effect of CST, which is a reasonable hypothesis. However, without direct measurements of ATP synthase activity (in both H9c2 myoblasts vs neonatal cardiomyocytes +/-CST), it is difficult to evaluate this interpretation. Thus, while the revision strengthens several parts of the study, the central mechanistic question regarding ATP synthase activity remains unanswered, and this limits the strength of the conclusions that can be drawn about CST's mode of action.

Response: We appreciate the reviewer's emphasis on directly measuring ATP synthase activity. As described above and now included in the revised manuscript (Fig. 8P–Q), we have performed ATP synthase (Complex V) activity assays using a NADH oxidation–based spectrophotometric approach.

These data demonstrate that CST treatment significantly increases ATP synthase catalytic activity in isolated mitochondria, accompanied by elevated mitochondrial membrane potential (Fig. 8O) and increased intracellular ATP levels (Fig. 8R). The direct enzymatic measurements therefore support our interpretation that CST enhances mitochondrial bioenergetic efficiency.

With respect to model-dependent effects, we agree that bioenergetic state may influence CST responsiveness. H9C2 myoblast-derived cells and neonatal cardiomyocytes differ in mitochondrial density, metabolic substrate preference, and oxidative capacity. The observed differences in Seahorse readouts likely reflect these intrinsic metabolic distinctions rather than absence of a CST effect on ATP synthase. Importantly, the direct enzymatic activity assay provides mechanistic evidence that CST enhances Complex V function.

We have revised the Discussion to clarify this point and to present the interpretation in a mechanistically balanced manner.
